# MYCN mediates cysteine addiction and sensitizes neuroblastoma to ferroptosis

**DOI:** 10.1038/s43018-022-00355-4

**Published:** 2022-04-28

**Authors:** Hamed Alborzinia, Andrés F. Flórez, Sina Kreth, Lena M. Brückner, Umut Yildiz, Moritz Gartlgruber, Dorett I. Odoni, Gernot Poschet, Karolina Garbowicz, Chunxuan Shao, Corinna Klein, Jasmin Meier, Petra Zeisberger, Michal Nadler-Holly, Matthias Ziehm, Franziska Paul, Jürgen Burhenne, Emma Bell, Marjan Shaikhkarami, Roberto Würth, Sabine A. Stainczyk, Elisa M. Wecht, Jochen Kreth, Michael Büttner, Naveed Ishaque, Matthias Schlesner, Barbara Nicke, Carlo Stresemann, María Llamazares-Prada, Jan H. Reiling, Matthias Fischer, Ido Amit, Matthias Selbach, Carl Herrmann, Stefan Wölfl, Kai-Oliver Henrich, Thomas Höfer, Andreas Trumpp, Frank Westermann

**Affiliations:** 1 Heidelberg Institute for Stem Cell Technology and Experimental Medicine, Heidelberg, Germany; 2 Institute of Pharmacy and Molecular Biotechnology, Heidelberg University, Heidelberg, Germany; 3 Division of Stem Cells and Cancer German Cancer Research Center and Center for Molecular Biology of the University of Heidelberg Alliance, Heidelberg, Germany; 4 Division of Theoretical Systems Biology, German Cancer Research Center, Heidelberg, Germany; 5 Hopp Children’s Cancer Center, Heidelberg, Germany; 6 Division of Neuroblastoma Genomics, German Cancer Research Center, Heidelberg, Germany; 7 Bioinformatics and Omics Data Analytics, German Cancer Research Center, Heidelberg, Germany; 8 Biomedical Informatics, Data Mining and Data Analytics, Augsburg University, Augsburg, Germany; 9 Metabolomics Core Technology Platform, University of Heidelberg, Heidelberg, Germany; 10 Proteome Dynamics, Max Delbrück Center for Molecular Medicine, Berlin, Germany; 11 Department of Immunology, Weizmann Institute of Science, Rehovot, Israel; 12 Department of Clinical Pharmacology and Pharmacoepidemiology, Heidelberg University Hospital, Heidelberg, Germany; 13 Berlin Institute of Health at Charité-Universitätsmedizin Berlin, Digital Health Center, Berlin, Germany; 14 Target Discovery Technologies, Bayer AG, Berlin, Germany; 15 Research & Development, Pharmaceuticals Division, Bayer AG, Berlin, Germany; 16 Division of Cancer Epigenomics, German Cancer Research Center, Member of the German Center for Lung Research, Heidelberg, Germany; 17 Translational Research to AdvanCe Therapeutics and Innovation in ONcology, The University of Texas MD Anderson Cancer Center, Houston, TX USA; 18 Experimental Pediatric Oncology, Children’s Hospital and Center for Molecular Medicine, Medical Faculty, University of Cologne, Cologne, Germany; 19 Charité-Universitätsmedizin Berlin, Berlin, Germany; 20 Health Data Science Unit, Medical Faculty University Heidelberg and BioQuant, Heidelberg, Germany; 21 German Cancer Consortium, Heidelberg, Germany; 22 Present Address: Department of Molecular and Cellular Biology, Harvard University, Cambridge, MA USA

**Keywords:** Cancer, Paediatric cancer, Cell death, Cancer therapy

## Abstract

Aberrant expression of MYC transcription factor family members predicts poor clinical outcome in many human cancers. Oncogenic MYC profoundly alters metabolism and mediates an antioxidant response to maintain redox balance. Here we show that MYCN induces massive lipid peroxidation on depletion of cysteine, the rate-limiting amino acid for glutathione (GSH) biosynthesis, and sensitizes cells to ferroptosis, an oxidative, non-apoptotic and iron-dependent type of cell death. The high cysteine demand of *MYCN*-amplified childhood neuroblastoma is met by uptake and transsulfuration. When uptake is limited, cysteine usage for protein synthesis is maintained at the expense of GSH triggering ferroptosis and potentially contributing to spontaneous tumor regression in low-risk neuroblastomas. Pharmacological inhibition of both cystine uptake and transsulfuration combined with GPX4 inactivation resulted in tumor remission in an orthotopic *MYCN*-amplified neuroblastoma model. These findings provide a proof of concept of combining multiple ferroptosis targets as a promising therapeutic strategy for aggressive *MYCN*-amplified tumors.

## Abstract (web summary)

Alborzinia et al. report that *MYCN*-amplified neuroblastoma undergoes ferroptosis in the absence of intracellular cysteine, suggesting a combination of cysteine depletion and concomitant GPX4 inactivation as a potential therapeutic approach.

## Main

Many human cancers rely on aberrant expression of MYC transcription factor family members to allow unhindered growth and proliferation; high expression levels are predictive of poor clinical outcome^1^. Aberrant MYC oncoprotein levels lead to gross transcriptional changes with hundreds, if not thousands, of upregulated and downregulated genes, which together drive various hallmark features of malignant cells. Pharmacological approaches to target aberrant MYC have largely failed. Therefore, MYC synthetic lethal interactions have been exploited for the development of therapeutic concepts to specifically target MYC-driven tumors, yet with limited success^2^. Remarkably, in view of MYC’s oncogenic activity, its potential to promote cell death apart from cell proliferation is paradoxical. Transgenic mouse models support a role for MYC for both tumor development but also spontaneous cell death depending on tissue type and context^3^. Both may ultimately be related to MYC’s profound influence on various aspects of cellular metabolism, with their interdependencies still poorly understood.

Childhood neuroblastoma, an embryonic tumor derived from progenitors of the sympathetic nervous system, is a paradigmatic model for MYC-driven cancers^4^. Amplified *MYCN* identifies a highly aggressive subtype associated with malignant progression and poor outcome despite intensive multimodal treatments. However, a substantial proportion of low-risk neuroblastomas with elevated *MYCN* expressed from a normal *MYCN* locus, particularly those arising in children younger than 18 months, regress spontaneously (stage 4S disease) by unknown mechanisms even when the disease is metastatic^5^. High-risk neuroblastomas lacking amplified *MYCN* harbor rearrangements of other *MYC* gene family members, *TERT* or alternative mechanisms of telomere lengthening (ALT) often associated with *ATRX* gene mutations, the latter subtype being incompatible with high *MYCN* or *MYC* expression^6,7^ (later referred to as MYC(N)). Beside these alterations linked to telomere maintenance mechanisms, mutations in *ALK* or other developmental genes lead to stalled differentiation and tumors composed of heterogeneous cell types resembling different states of the normal neuroendocrine differentiation trajectories. The spectrum of cell types ranges from differentiated over undifferentiated adrenergic-to-mesenchymal cell types (triggered by adrenergic-to-mesenchymal transition), where malignant progression, therapy resistance and disease relapse are strongly associated with undifferentiated cell types.^8,9^

*MYCN*-amplified neuroblastoma cells, like other MYC-driven cancer cells, have been found to be addicted to the amino acid glutamine (Gln), the absence of which causes growth arrest or apoptosis^10^. More recently, reports showed that neuroblastoma cells are also addicted to iron and are sensitized to ferroptosis^11,12^, a new iron-dependent oxidative form of cell death associated with lipid peroxidation and insufficient capacity to eliminate lipid peroxides^13^. Whereas the apoptosis pathway of regulated cell death is often genetically or epigenetically impaired in primary high-risk and relapsed neuroblastomas^14^, there is only limited knowledge of how metabolic rewiring as a consequence of aberrant *MYCN* controls the liabilities of fast-proliferating malignant cells that are confronted with accumulating reactive oxygen species (ROS).

In this study, by performing single amino acid deprivations in high *MYCN* and low *MYCN* neuroblastoma cells, we discovered strong dependency of high *MYCN* cells on the amino acid cysteine. Using functional *MYCN* synthetic lethal metabolic and genetic screens, we further identified cyst(e)ine deprivation and glutathione peroxidase 4 (GPX4) inhibition as selective liabilities in *MYCN*-amplified neuroblastomas. Combined targeting of cystine uptake, cysteine synthesis via transsulfuration and GPX4 in an orthotopic neuroblastoma model strongly reduced tumor growth in vivo. Multi-omics profiling identified multiple cell type-specific and MYCN-regulated mechanisms inhibiting ferroptosis in adrenergic and mesenchymal neuroblastoma cells. Taken together, our study uncovered mechanisms crucial to ferroptosis escape in *MYCN*-amplified neuroblastomas; simultaneous inhibition of those mechanisms led to tumor regression in vivo.

## Results

### Cystine deprivation induces MYCN-dependent ferroptosis

**Fig. 1:**
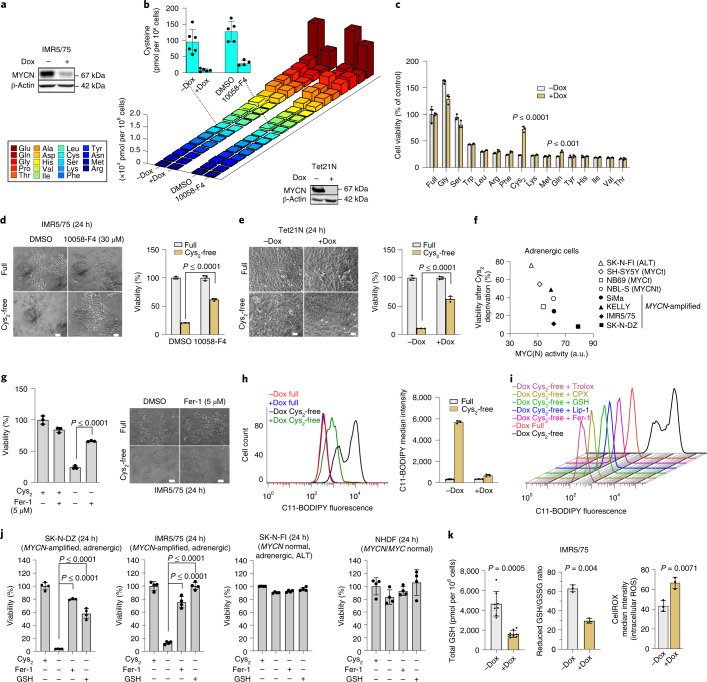
Cystine addiction in *MYCN*-expressing neuroblastoma cells. **a**, Representative western blot of IMR5/75 neuroblastoma cells on *MYCN* knockdown using Dox (expression: −Dox, high; +Dox, low); the experiment was replicated three times. **b**, Intracellular amino acid quantification after *MYCN* inhibition for 96 h (+Dox, *n* = 5 samples and −Dox, *n* = 6 samples, or 10058-F4, inhibiting MYCN–MAX binding, *n* = 4 samples and dimethyl sulfoxide (DMSO)-treated, *n* = 5 samples). Data represent the mean ± s.e.m. The experiment was replicated three times. **c**, Standardized viability of IMR5/75 after single amino acid depletions (48 h). Data represent the mean ± s.e.m.; *n* = 3 samples. The experiment was replicated three times. **d**,**e**, Cellular responses to Cys_2_ deprivation in high *MYCN* (−Dox) and low *MYCN* (+Dox) state in IMR5/75 (**d**) and Tet21N (**e**) cells; the mean viability of cells was standardized to untreated (full medium) and representative western blot of neuroblastoma Tet21N cells. Data represent the mean ± s.e.m.; *n* = 3 samples. The experiment was replicated three times. Images of the cells are shown on the left. Scale bar, 50 μm. **f**, Sensitivity to Cys_2_ deprivation versus level of MYC(N) activity in a panel of neuroblastoma cell lines. **g**, Relative viability (survival of compound-treated cells divided by survival of vehicle-treated cells) of IMR5/75 cells after Cys_2_ deprivation for 72 h in the presence or absence of Fer-1. Data represent the mean ± s.e.m.; *n* = 3 samples. The experiment was replicated three times. Images of the cells are shown on the left. Scale bar, 50 μm. **h**, Analysis of lipid peroxidation in Cys_2_-deprived high or low *MYCN* IMR5/75 cells (*n* = 3 samples; the experiment was replicated 3 times). **i**, Analysis of lipid peroxidation in Cys_2_-deprived high or low *MYCN* IMR5/75 cells in the presence or absence of Trolox, CPX, Lip-1, GSH or Fer-1. The experiment was replicated three times. **j**, Relative viability of SK-N-DZ, IMR5/75, SK-N-FI and normal human dermal fibroblasts (NHDFs) after Cys_2_ deprivation in the presence or absence of GSH and Fer-1. *n* = 4 samples. The experiment was replicated three times. **k**, Quantification of total intracellular GSH (*n* = 8 samples) levels and the reduced GSH/GSH disulfide (GSSG) ratio in IMR5/75 cells (*n* = 3 samples). Analysis of intracellular ROS levels using CellROX staining and flow cytometry in IMR5/75 cells in high *MYCN* (−Dox) and low *MYCN* (+Dox) state (*n* = 3 samples). Data represent the mean ± s.e.m. The experiment was replicated three times. Statistical analysis was performed using a two-tailed Student’s *t*-test. Source data

**Extended Data Fig. 1:**
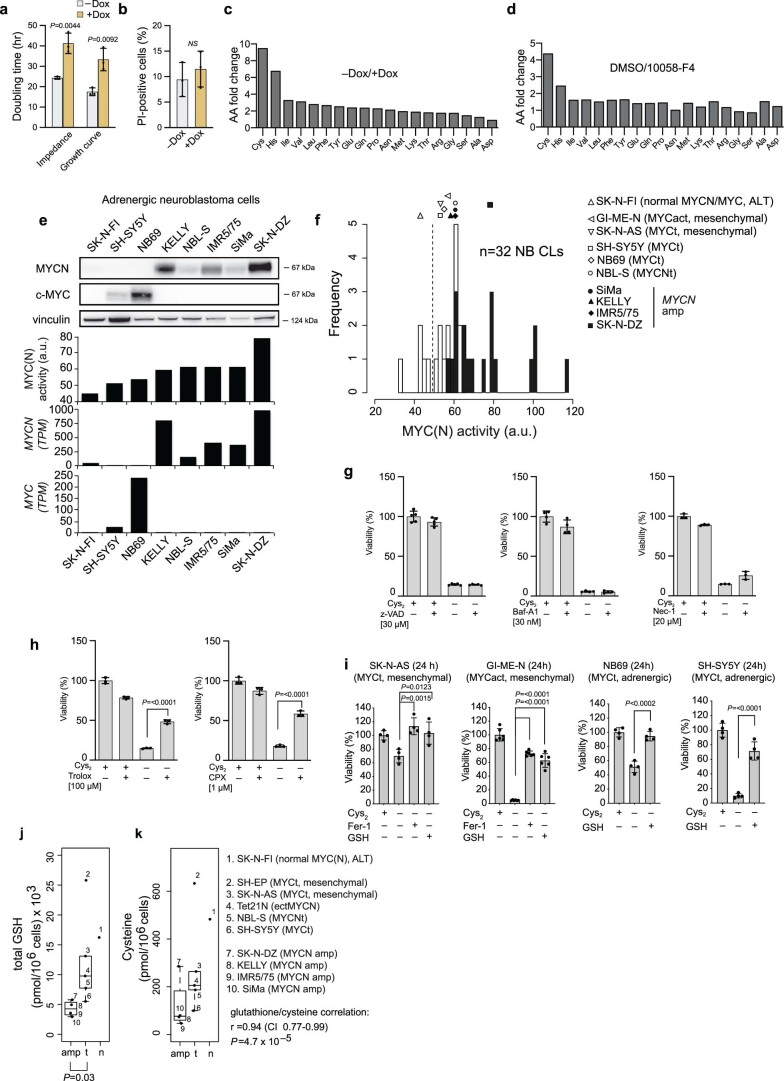
Cell lines phenotypic data. **a**, Doubling time calculation from exponential growth curve quantified by FACS and impedance measured using the RCTA xCELLigence system for high-MYCN (–Dox) and low-MYCN ( + Dox) IMR5/75 cells. Cell proliferation curves of exponentially growing cells (*n* = 3 samples, experiment replicated 3 times). **b**, Viability quantification using FACS and propidium iodide staining in exponentially growing cells: high *MYCN* (–Dox) vs. low *MYCN* ( + Dox) (*n* = 3 samples, experiment replicated 3 times). **c**, **d**, Fold changes of intracellular amino acid levels after MYCN inhibition (+Dox or 10058-F4, inhibiting MYCN/MAX binding, 96 h). **e**, MYC(N) RNA, protein expression (experiment replicated 3 times) and **f**, ‘MYC(N) activity’ score^39^ for a panel of adrenergic neuroblastoma cell lines. MYC(N) activity score of 32 neuroblastoma (NB) cell lines (CLs) having different *MYC(N*) genetic status (t = translocation; amp = amplification; act = activated due to unknown mechanism). **g**, Relative viability of IMR5/75 cells after Cys_2_ deprivation in the presence or absence of Inhibitors of apoptosis (z-VAD FMK, *n* = 5 samples), autophagy (Bafilomycin A1, Baf-A1, *n* = 4 samples,) and necroptosis (RIPK1 inhibitor, Necrostatin-1, Nec-1, *n* = 3 samples) and **h**, Trolox, or intracellular iron chelator ciclopirox olamine (CPX); *n* = 3 samples, experiment replicated 3 times. **i**, Relative viability of neuroblastoma cell lines after Cys_2_ deprivation in the presence or absence of ferrostatin-1 (Fer-1) or glutathione (GSH); *n* = 4 samples for SK-N-AS, NB69 and SH-SY5Y cell lines and *n* = 6 samples for GI-ME-N cell line; experiment replicated 3 times. **j**, Total GSH and **k**, cysteine (Cys) levels in neuroblastoma cells with amplified *MYCN*, *MYC(N)* translocations or normal *MYC(N)* status; Wilcoxon rank-sum test; center line indicates the median value, lower and upper hinges represent the 25th and 75th percentiles, respectively, and whiskers denote 1.5× interquartile range. Each dot corresponds to one sample; *p*-values as indicated. Source data

First, we analyzed the interplay of oncogenic MYCN activity with amino acid metabolism. Downregulating MYCN in the *MYCN*-amplified IMR5/75 neuroblastoma cell model^15^ (approximately 65% reduction; Fig. 1a) slowed cell proliferation without inducing cell death (Extended Data Fig. 1a,b) and reduced the intracellular pools of all amino acids (Fig. 1b). Most prominently, cysteine was reduced nearly tenfold (Extended Data Fig. 1c). Inhibiting MYCN binding to Myc-associated factor X (MAX) using 10058-F4 (ref. ^16^) yielded similar results (Fig. 1b and Extended Data Fig. 1d). These data show that high MYCN levels are associated with high levels of cellular cysteine, probably mediated by increased synthesis and/or uptake from the microenvironment. Systematic depletion of individual amino acids from the growth medium impaired cell viability in both high *MYCN* and low *MYCN* cells in most cases (Fig. 1c). However, in line with recent reports^11,12^, cells with high *MYCN* expression exhibited stronger dependency on cystine imported by cystine/glutamate-exchange transporter x_c_^−^ and readily reduced to two cysteine molecules intracellularly. Cystine deprivation caused robust cell death in high *MYCN* cells, which was largely prevented by downregulation of *MYCN* expression (Fig. 1c) or inhibition of MYCN–MAX binding (Fig. 1d). Overexpressing *MYCN* in *MYCN* diploid cells (Tet21N neuroblastoma cell model^17^), rendered these cells highly vulnerable to cystine deprivation (Fig. 1e). Neuroblastoma cell lines with intermediate *MYCN* or *MYC* levels caused by gene translocations are known to exist. We inferred MYC(N) activity by target gene expression score^5^, which agrees with transcript and protein MYC(N) levels (Extended Data Fig. 1e,f). Cell death after cystine deprivation increased with MYC(N) activity score, being virtually absent in cell lines immortalized by alternative telomere lengthening and lacking MYC(N) aberrations and peaking in *MYCN*-amplified cell lines with the highest activity scores (Fig. 1f). These data demonstrate that oncogenic MYC(N) expression is associated with cysteine addiction, with cysteine reduction resulting in massive cell death in an MYC(N)-dependent manner.

Cell death in the cystine-deprived high *MYCN* cells was not abrogated by the inhibition of (1) caspases to prevent apoptosis, (2) lysosomal function to downregulate autophagy or (3) receptor-interacting serine/threonine-protein kinase 1 (RIPK1) to prevent necroptosis (Extended Data Fig. 1g). Availability of cystine/cysteine is the rate-limiting step in GSH synthesis counteracting ROS^18^, which prevents the execution of ferroptosis, an oxidative, non-apoptotic and iron-dependent form of regulated cell death caused by ROS-mediated lipid peroxidation^13,19,20^. Indeed, ferrostatin-1 (Fer-1), a specific inhibitor of ferroptosis, or a lipophilic antioxidant or an intracellular iron chelator such as ciclopirox olamine (CPX) averted death in cystine-deprived high *MYCN* neuroblastoma cells (Fig. 1g and Extended Data Fig. 1h). Monitoring ROS formation by flow cytometry using the lipid peroxidation sensor, C11-BODIPY, showed that cystine deprivation dramatically increased cellular lipid peroxidation selectively in high *MYCN* cells (Fig. 1h). Inhibiting ferroptosis with lipophilic antioxidants or an intracellular iron chelator inhibited lipid peroxidation in cystine-deprived high *MYCN* cells (Fig. 1i). Hence, when deprived of cystine, high *MYCN* neuroblastoma cells exhibited lipid peroxidation and died via ferroptosis.

Addition of GSH to the cystine-free medium also prevented lipid peroxidation in *MYCN*-amplified cells similar to Fer-1 (Fig. 1i) and rescued cell viability similar to Fer-1 (Fig. 1j and Extended Data Fig. 1i), suggesting that high GSH levels protect against ferroptosis. Consistent with their higher sensitivity to ferroptosis, *MYCN*-amplified neuroblastoma cells had lower baseline GSH and cysteine levels compared to cell lines with lower MYC(N) activity scores (Extended Data Fig. 1j,k). However, on downregulation of *MYCN* in *MYCN*-amplified cells, intracellular GSH levels were reduced threefold, the reduced-to-oxidized GSH ratio was halved and intracellular ROS levels increased (Fig. 1k), suggesting that high *MYCN* expression increases GSH synthesis and ROS clearance. Overall, these results indicate that although oncogenic *MYCN* activates the production of GSH, it is maintained at a low steady state level due to its rapid consumption in fast-proliferating cells.

**Fig. 2:**
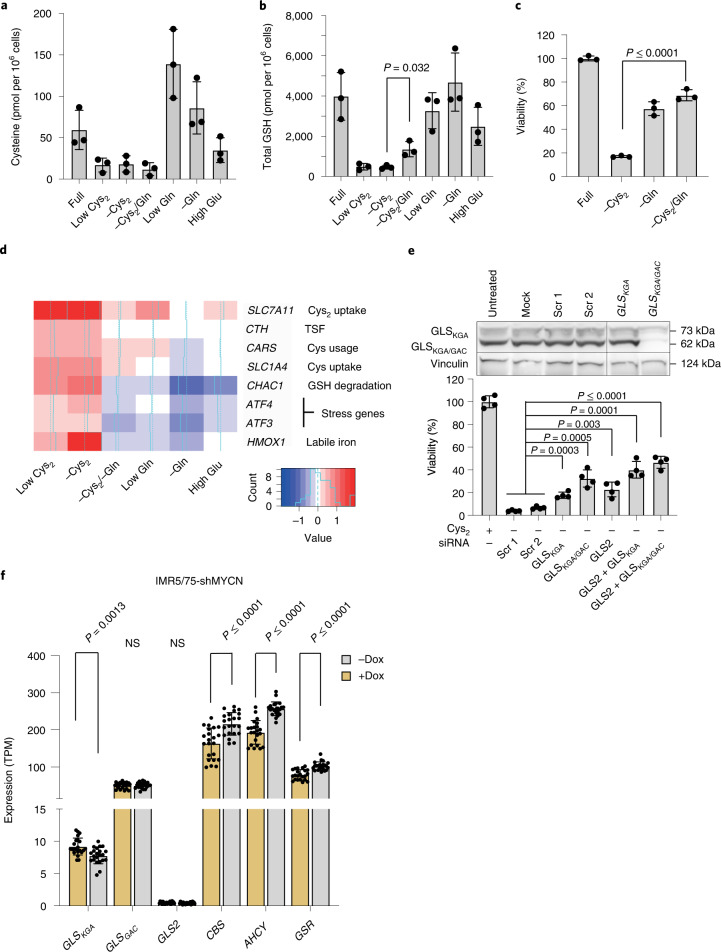
Gln is required for ferroptosis in high *MYCN* cells. **a**–**c**, Cysteine (**a**), total GSH levels (**b**) and cell viability (**c**) on Cys_2_ and Gln deprivation. Data represent the mean ± s.e.m.; *n* = 3 samples. The experiment was replicated three times. **d**, RNA-seq of IMR5/75 cells on treatment with the conditions indicated in the figure. *n* = 3 samples. **e**, siRNA-mediated GLS_KGA/GAC_ knockdown (72 h) on Cys_2_ deprivation (24 h) in IMR5/75 cells. Representative western blots of each isoform. Data represent the mean ± s.e.m.; *n* = 4 samples. The experiment was replicated three times. **f**, Expression of glutaminolysis genes (mitochondrial *GLS*_*GAC*_; cytosolic *GLS*_*KGA*_ and *GLS2*) compared with MYCN-regulated genes (*CBS*, *AHCY*, *GSR*) in high *MYCN* and low *MYCN* IMR5/75 cells. TPM, transcripts per million. Data represent the mean ± s.e.m. The statistical analysis was performed using a two-tailed Student’s *t*-test. Source data

The only other amino acid showing a selective dependency on *MYCN* was Gln, confirming previous reports^10,21^, although this was significantly less pronounced (Fig. 1c). Interestingly, cysteine and Gln (when converted to glutamate) are two important GSH precursors. Depleting Gln in addition to cystine partially restored GSH levels probably because of less lipid peroxidation^22^ and rescued cell viability (Fig. 2a–c). Gln/cystine deprivation prevented the induction of ferroptosis-promoting genes (*CHAC1*, *HMOX1*) and reduced transcriptional activation of genes protecting against cellular stress (*ATF4*, *ATF3*) shown to be activated on cystine depletion^23^ (Fig. 2d). Besides Gln depletion, inhibiting glutaminolysis should prevent ferroptosis^24^. Indeed, knockdown of either glutaminase isoform, GLS_KGA/GAC_, averted ferroptosis confirming that glutaminolysis is required to induce ferroptosis in cystine-deprived high *MYCN* cells (Fig. 2e**)**. However, since *GLS*_*KGA*_ is repressed by *MYCN* (Fig. 2f), *GLS*_*GAC*_ appears to be the main glutaminase isoform involved in these cells.

### Identification of ferroptosis genes in *MYCN*-amplified cells

**Fig. 3:**
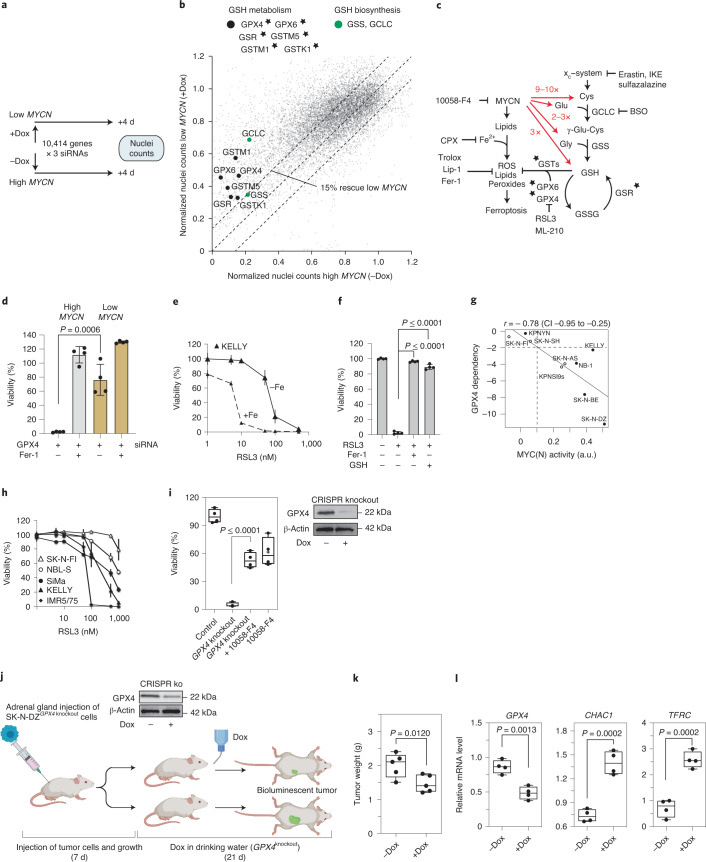
Inhibition of *GPX4* is synthetic lethal with high *MYCN*. **a**, *MYCN* synthetic lethal druggable genome-wide siRNA screening approach in IMR5/75 cells. **b**, Effects of individual siRNAs (gray dots): high *MYCN* versus low *MYCN*, including key players (median of two or three siRNAs) of the top *MYCN* synthetic lethal hits (single star symbol) of GSH metabolism (black) and biosynthesis (green). **c**, *MYCN* effects on lipid peroxide formation and intracellular amino acid levels (fold changes shown in red), the x_c_^−^ system (Cys_2_ uptake), the two-step biosynthesis of GSH and GSH metabolism; the single star symbol marks the top *MYCN* synthetic lethal hits of GSH metabolism, with an FDR of 0.2. The action of ferroptosis inhibitors (CPX, Fer-1, Lip-1, Trolox and 10058-F4), class I (erastin, IKE, sulfasalazine), class II ferroptosis inducers (RSL3, ML-210) and the GSH biosynthesis inhibitor buthionine sulfoximine as indicated. **d**, siRNA *GPX4* knockdown in the presence or absence of Fer-1. Data represent the mean ± s.e.m.; *n* = 4 samples. The experiment was replicated three times**. e**, Relative viability (survival of compound-treated cells divided by survival of vehicle-treated cells) of the KELLY cell line after cotreatment with iron sucrose (25 µg ml^−1^) and RSL3. Data represent the mean ± s.e.m.; *n* = 3 samples. The experiment was replicated three times. **f**, Relative viability of IMR5/75 cells treated with RSL3 in the presence or absence of Fer-1 or GSH. *n* = 4 samples. The experiment was replicated three times. **g**, DRIVE database^27^ (RSA values, Pearson’s correlation, *P* = 0.01; filled circle, *MYCN*-amplified; white circle, *MYCN*-non-amplified). **h**, Cellular responses of neuroblastoma cell lines to 72 h of RSL3 treatment: cells with *MYCN* amplification (black symbols), moderate *MYCN* expression (white circle) and lack thereof (white triangle). Data represent the mean ± s.e.m.; *n* = 3 samples. The experiment was replicated three times. **i**, Dox-inducible *GPX4* CRISPR–Cas9 knockout in a 3D model with *MYCN*-amplified SK-N-DZ cells in the presence or absence of MYCN–MAX inhibition. Data represent the mean ± s.e.m. Right: representative western blot; *n* = 4 samples. The experiment was replicated three times. **j**, Orthotopic mouse neuroblastoma model allowing CRISPR–Cas9-mediated *GPX4* deletion. Panel created with BioRender. **k**, Tumor weight after *GPX4* knockout (+Dox) (*n* = 5 mice per group). A representative western blot for CRISPR–Cas9-mediated *GPX4* deletion is shown. **l**, Elevated messenger RNA expression of the ferroptosis markers *CHAC1* and *TFRC*. Data represent the mean ± s.e.m.; *n* = 4 samples from each group. Statistical analysis was performed using a one-tailed Student’s *t*-test for the in vivo experiments and a two-tailed Student’s *t*-test for the in vitro experiments. Box plots: the center line indicates the median value, the lower and upper hinges represent either the 25th and 75th percentiles or the minimum and maximum points and the whiskers denote 1.5× the interquartile range (IQR). Each dot corresponds to one sample; one-sided Student’s *t*-test; *P* values as indicated. Source data

**Extended Data Fig. 2:**
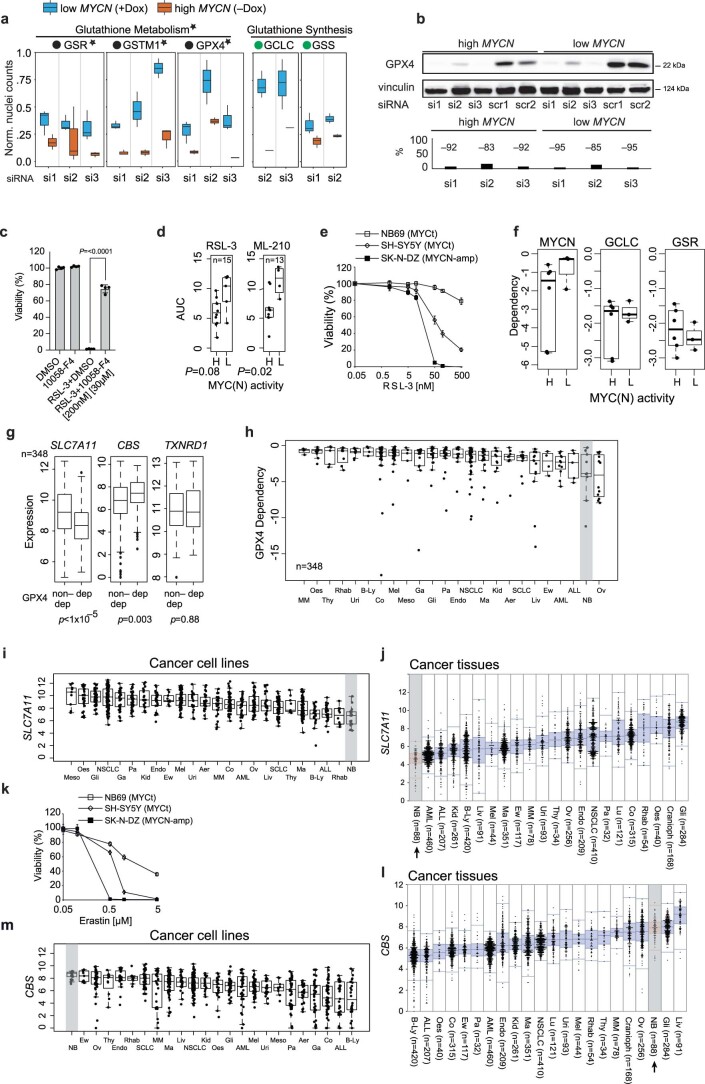
Additional screen targets and expression data. **a**, MYCN synthetic lethal screen hits. MYCN effects on the two-step biosynthesis of GSH and GSH metabolism (^★^top MYCN synthetic lethal hits of GSH metabolism, false discovery rate of 0.2) (median of 2-3 siRNAs). **b**, Western Blot and quantification of siRNA-mediated *GPX4* knockdown (96 h) in Tet21N cells with doxycycline-regulatable MYCN (experiment replicated 3 times). **c**, Relative viability (survival of compound-treated cells divided by survival of vehicle-treated cells) of IMR5/75 cell line after treatment with RSL-3 in the presence or absence of MYC inhibitor (10058-F4) (*n* = 4 samples, experiment replicated 3 times) and **d**, Response to GPX4 inhibitors (RSL-3, ML-210) (CTRPv2 database^54^); H = high and L = low MYC(N) activity; Wilcoxon rank-sum test. **e**, Sensitivity to RSL-3-induced ferroptosis in *MYCN*-amplified neuroblastoma cell lines (*n* = 4 samples, experiment replicated 3 times). **f**, MYC(N) activity correlation with GCLC or GSR dependency in nine neuroblastoma cell lines (DRIVE database^39^). **g**, Low expression of *SLC7A11* and high expression of *CBS* associated with GPX4 dependency in 384 cancer cell lines (DRIVE database^39^). *TXNRD1* expression dependent on GPX4 knockdown sensitivity in 384 cancer cells lines (Wilcoxon rank-sum test). **h**, GPX4 dependency in 348 cancer cell lines grouped according to cancer entities (DRIVE database^39^). **k**, Relative viability of NB69, SH-SY5Y and SK-N-DZ cell lines after treatment with erastin. *n* = 3 samples, experiment replicated 3 times. **i**, **j, l, m**, Expression of *SLC7A11* or *CBS* in cell lines (*CCLE* database, n = 917^55^) or tissues (R2 database^56^). Box plots: center line indicates the median value, lower and upper hinges represent the 25th and 75th percentiles, respectively, and whiskers denote 1.5× interquartile range. Each dot corresponds to one sample; Wilcoxon rank-sum test; *p*-values as indicated. Source data

To systematically identify the cellular vulnerabilities associated with high *MYCN* expression, we performed an unbiased synthetic lethal small interfering (siRNA) screen using high/low *MYCN* IMR5/75 cells (Fig. 3a). Among the top hits that reduced the survival of high *MYCN* cells were enzymes involved in GSH metabolism that detoxify lipid peroxides (Fig. 3b,c, Extended Data Fig. 2a and Supplementary Table 1). Namely, inhibition of *GSR*, *GPX4*, *GPX6* or *GSTM1, GSTM5* and *GSTK1* (Fig. 3b and Extended Data Fig. 2a) caused a selective reduction in viability in high *MYCN* cells. Knockdown of either of the two enzymes catalyzing GSH biosynthesis, glutamate–cysteine ligase catalytic subunit (GCLC) and glutathione synthetase (GSS) also showed synthetic lethality with high *MYCN* state (Fig. 3b and Extended Data Fig. 2a).

One of the screening hits, GPX4, is an anti-ferroptotic selenoprotein that protects against lipid peroxidation using GSH^25,26^. siRNA-mediated GPX4 knockdown induced ferroptosis in high *MYCN* cells, which was rescued by Fer-1 (Fig. 3d and Extended Data Fig. 2b). Similarly, we observed reduction of viability using the GPX4 inhibitor RSL3, enhanced by supplementing iron and rescued by Fer-1 or GSH (Fig. 3e,f) or simultaneous MYC(N) activity inhibition using 10058-F4 (Extended Data Fig. 2c). The dependency of cell viability on GPX4 activity increased with MYC(N) activity across our broader cell line panel (Fig. 3g,h and Extended Data Fig. 2d,e) unlike GCLC or GSR, which exhibited no dependency **(**Extended Data Fig. 2f). Next, we studied whether the effect of GPX4 knockdown was linked to cystine uptake and metabolic gene expression in 348 cancer cell lines^27^. Cells dependent on GPX4 showed low expression levels of *SLC7A11* of the x_c_^−^ system, importing cystine in exchange for glutamate (Extended Data Fig. 2g). Neuroblastoma cell lines were, after ovarian cancer cell lines, the second most dependent on GPX4 (Extended Data Fig. 2h).

To therapeutically investigate the role of *GPX4* inhibition in vivo, we used an inducible CRISPR–Cas9 system to eliminate *GPX4* (*GPX4* knockout). In the *MYCN*-amplified SK-N-DZ three-dimensional (3D) cell culture model, *GPX4* knockout induced ferroptotic cell death. This was rescued by simultaneously treating cells with the 10058-F4 MYC–MAX inhibitor (Fig. 3i). We then generated an orthotopic mouse model for human neuroblastoma by transplanting these SK-N-DZ neuroblastoma cells orthotopically into the adrenal gland and allow the cells expressing native GPX4 levels to develop into small tumors (Fig. 3j). Subsequently, the CRISPR–Cas9 *GPX4* knockout was induced by doxycycline (Dox) treatment 7 d after transplantation. Although this resulted in a significant reduction in tumor weight compared to controls (Fig. 3k), it was not sufficient for tumor eradication as also shown by others^28^. Ferroptosis markers such as *CHAC1* and *TFRC*^29^ increased despite only partial reduction of *GPX4* transcripts, suggesting activation of ferroptosis in vivo (Fig. 3l). In summary, our data show that GPX4 partially protects high *MYCN* neuroblastoma cells from ferroptosis in vitro and in an orthotopic neuroblastoma mouse model.

**Fig. 4:**
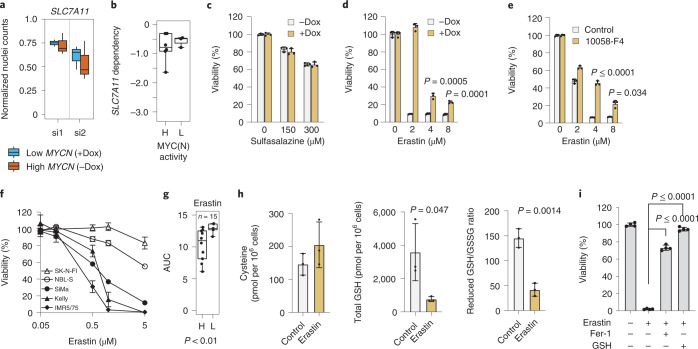
Inhibition of cystine uptake by SLC7A11 induces ferroptosis in high *MYCN* cells. **a**, SLC7A11 knockdown effect in the IMR5/75 cell line. **b**, Correlation between MYC(N) activity and sensitivity to *SLC7A11* repression (DRIVE^27^). **c**, Relative viability (survival of compound-treated cells divided by survival of vehicle-treated cells) of both high and low *MYCN* IMR5/75 cells after treatment with sulfasalazine. **d**,**e**, Treatment with erastin for 72 h with MYCN knockdown (**d**) and MYC/MAX inhibitor (**e**). Data represent the mean ± s.e.m.; *n* = 3 samples. The experiment was replicated three times. **f**,**g**, Cellular responses of neuroblastoma cell lines to 72 h of erastin treatment. Data represent the mean ± s.e.m.; *n* = 3 samples. The experiment was replicated three times) (**f**) and confirmed by the CTRPv2 (ref. ^57^) data (**g**). AUC, area under the curve. **h**, Cysteine and GSH levels and reduced GSH/GSSG ratio in IMR5/75 cells treated with erastin. Data represent the mean ± s.e.m.; *n* = 3 samples. The experiment was replicated three times. **i**, Relative viability of IMR5/75 cells treated with erastin in the presence or absence of Fer-1 or GSH (72 h). Data represent the mean ± s.e.m.; *n* = 4 samples. The experiment was replicated three times. Box plots: the center line indicates the median value, the lower and upper hinges represent the 25th and 75th percentiles, respectively and the whiskers denote 1.5× the IQR. Each dot corresponds to one sample; Wilcoxon rank-sum test, *P* values as indicated. Source data

Both neuroblastoma cell lines and primary neuroblastomas had the lowest *SLC7A11* expression compared to other tumor entities (Extended Data Fig. 2i,j). This suggests that the sensitivity of neuroblastoma to cystine depletion and GPX4 inhibition is due, at least in part, to reduced uptake of cystine^18^. SLC7A11 knockdown or inhibition by sulfasalazine moderately reduced viability regardless of *MYCN* level (Fig. 4a–c) as shown recently^12^. Erastin, a more potent SLC7A11 inhibitor^23^, selectively induced ferroptosis in the high *MYCN* state (Fig. 4d), which was partially rescued by additional inhibition of MYC(N) activity (Fig. 4e). Erastin-induced cell death increased with MYC(N) activity score in neuroblastoma cell lines (Fig. 4f,g and Extended Data Fig. 2k), was associated with decreased GSH and reduction of the GSH reduced-to-oxidized ratio (Fig. 4h) and could be prevented by providing either GSH or Fer-1 (Fig. 4i). Taken together, cystine uptake by SLC7A11 maintains part of the necessary cysteine for GSH production to protect against ferroptosis.

### MYCN induces transsulfuration and prevents ferroptosis

**Fig. 5:**
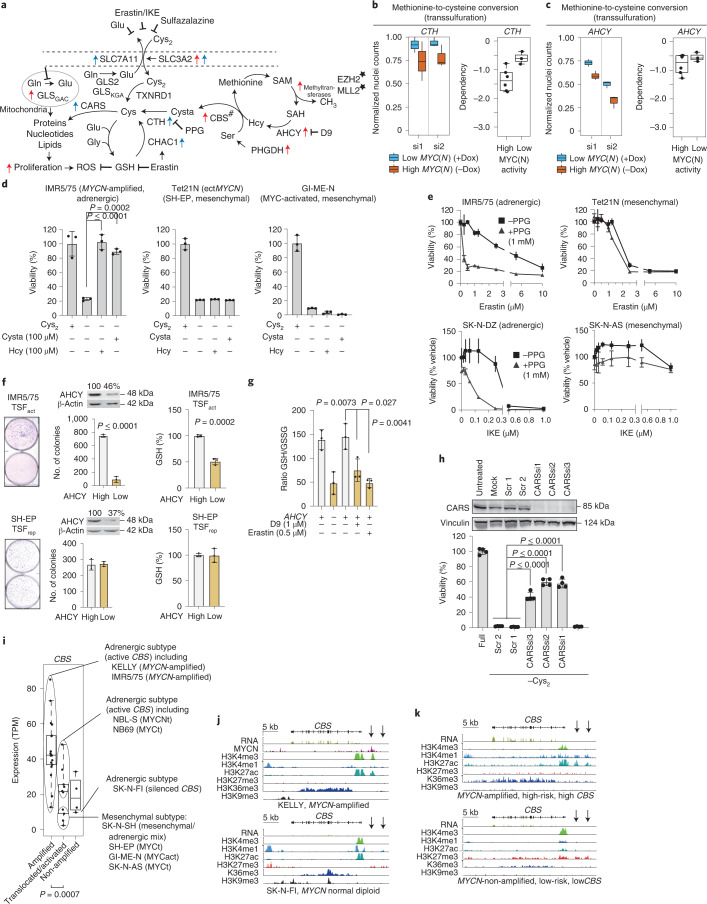
MYCN activates transsulfuration genes controlling methionine-to-cysteine conversion in *MYCN*-amplified cells. **a**, Illustration of Cys_2_ uptake/metabolism, glutaminolysis, methionine cycle and transsulfuration (^#^). The single star indicates the top hits from the MYCN siRNA screen; the red arrows indicate genes transcriptionally activated by MYCN in IMR5/75 (FDR = 0.001, using likelihood ratio testing); the blue arrows indicate genes transcriptionally activated by Cys_2_ deprivation in IMR5/75 cells. **b**,**c**, siRNA screen in the IMR5/75 cell line and MYCN synthetic lethality with knockdown of key transsulfuration genes (two individual siRNAs) and MYC(N) activity correlation with *CTH* (**b**) and *AHCY* (**c**). **d**, Relative viability of IMR5/75, Tet21N and GI-ME-N cells after Cys_2_ deprivation for 48 h in the presence or absence of Hcy or Cysta. Data represent the mean ± s.e.m.; *n* = 3 samples. The experiment was replicated three times. ectMYCN, ectopic MYCN. **e**, Relative viability (survival of compound-treated cells divided by survival of vehicle-treated cells) of IMR5/75, SK-N-DZ and *MYCN*-expressing mesenchymal Tet21N and *MYC*-expressing SK-N-AS cells after cotreatment with PPG and erastin or IKE. Data represent the mean ± s.e.m.; *n* = 3 samples. The experiment was replicated three times. **f**,**g**, Colony formation, GSH synthesis (**f**) and reduced GSH/GSSG ratio (**g**) in IMR5/75 and SH-EP cells on *AHCY* inhibition (by knockdown or using small molecule D9). Representative western blots are shown in the figure. Data represent the mean ± s.e.m.; *n* = 3 samples. The experiment was replicated three times. **h**, Relative viability of IMR5/75 cells after siRNA-mediated *CARS* knockdown (72 h) and Cys_2_ deprivation (24 h). Representative western blots are shown in the figure. Data represent the mean ± s.e.m.; *n* = 4 samples. The experiment was replicated three times. **i**, *CBS* expression in 32 neuroblastoma cell lines (RNA-seq) categorized using *MYCN*-amplified, *MYCN*/*MYC*-translocated/activated cell lines and MYC(N) non-expressors. MYCNt/MYCt, MYCN or MYC translocation; MYCact, activated MYC due to unknown mechanism. **j**,**k**, Bimodal *CBS* expression for *MYCN*/*MYC*-translocated/activated cell lines along with adrenergic and mesenchymal cell type mRNA expression (RNA-seq) and input-normalized read counts of histone modifications and MYCN (ChIP–seq) at the *CBS* locus in representative *MYCN*-amplified and *MYCN*-non-amplified cells (**j**) and tumors (**k**). Box plots: the center line indicates the median value, the lower and upper hinges represent the 25th and 75th percentiles, respectively and the whiskers denote 1.5× the IQR. Each dot corresponds to one sample; Wilcoxon rank-sum test; *P* values as indicated. Source data

**Extended Data Fig. 3:**
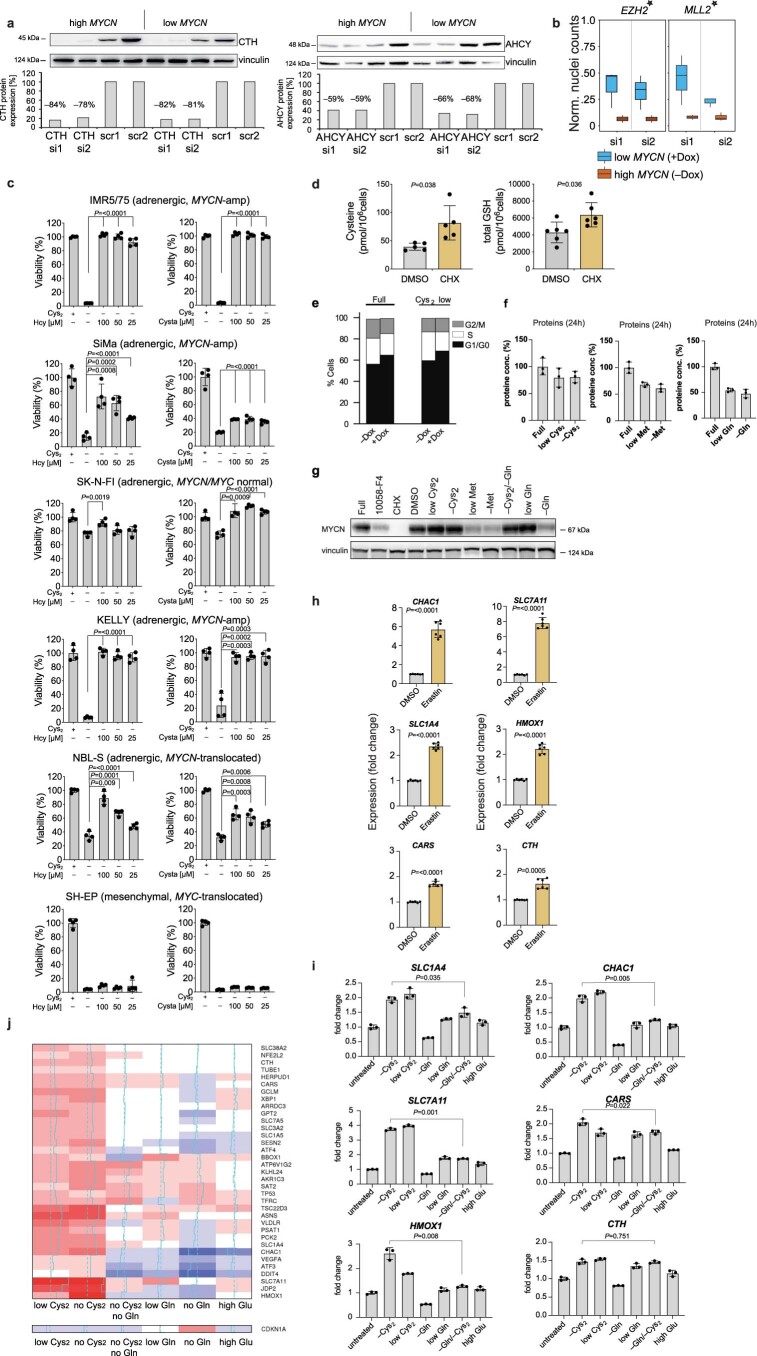
Quantification of transsulfuration activity. **a**, Western Blot and quantification of CTH or AHCY protein expression upon siRNA-mediated knockdown in IMR5/75 high *MYCN* or low *MYCN* cells. Experiment replicated 3 times. **b**, Two methyltransferases that may indirectly increase Hcy levels are synthetic lethal with high MYCN (MYCN siRNA screen; ^★^false discovery rate of 0.2); center line indicates the median value of 2-3 siRNAs, lower and upper hinges represent the 25th and 75th percentiles, respectively, and whiskers denote 1.5× interquartile range. **c**, Relative viability (survival of compound-treated cells divided by survival of vehicle-treated cells) of adrenergic and mesenchymal neuroblastoma cell lines after Cys_2_ deprivation in the presence or absence of Cysta or Hcy. *n* = 4 samples, experiment replicated 3 times. **d**, Impact of protein synthesis inhibition by cycloheximide (CHX) on cysteine (*n* = 5 samples) and glutathione level (*n* = 6 samples and CHX, 100 ng/ml, 24 h and experiment replicated 3 times). **e**, Cell cycle analysis of *MYCN*-amplified IMR5/75 cells (24 h) after Cys_2_ depletion (representative measurement of 3 replicates). **f**, Analysis of protein synthesis in *MYCN*-amplified IMR5/75 cells (24 h) after low levels or complete depletion of Cys_2_, methionine (Met) or glutamine (Gln) in the culture medium (*n* = 3 samples, experiment replicated 3 times). **g**, MYCN protein expression (by western blot) upon MYCN-MAX inhibition (10058-F4), protein synthesis inhibition by cycloheximide (CHX, 100 ng/ml) and low levels or complete depletion of Cys_2_, methionine (Met) or glutamine (Gln) in the culture medium after 8 h. Experiment replicated 3 times. **h**, **i**, **j**, RNA-seq and RT-qPCR of IMR5/75 cells 24 h following Cys, Gln depletion and Glu excess or erastin treatment and regulation of ferroptosis-related genes (*n* = 6 samples in **h**, *n* = 3 samples in **i**; experiment replicated 3 times). Statistical analysis was performed using Student’s two-tailed t-test. Source data

Cellular cysteine can also be produced in some cell types by transsulfuration^30,31^. In this process, homocysteine (Hcy), an intermediate of the methionine cycle, and serine are combined to form cystathionine (Cysta), which is further converted to cysteine^32^ (Fig. 5a). GPX4 dependency in cancer cell lines was associated with enhanced expression of cystathionine beta-synthase (*CBS*), the rate-limiting enzyme for transsulfuration (Extended Data Fig. 2g), with neuroblastoma being among the cancer entities with the highest *CBS* expression (Extended Data Fig. 2m,l). We hypothesized that transsulfuration provides a cysteine source for neuroblastoma cells preventing ferroptosis in cystine deprivation conditions. Cystathionine gamma-lyase (CTH), converting Cysta to cysteine, and S-adenosyl-L-homocysteine hydrolase (AHCY), synthesizing Hcy for transsulfuration, show synthetic lethality with high *MYCN* (Fig. 5b,c and Extended Data Fig. 3a), as are two methyltransferases that feed into Hcy production (Extended Data Fig. 3b). Supplementing cystine-deprived cells with either Hcy or Cysta prevented ferroptosis in all adrenergic neuroblastoma cell lines tested with high or intermediate oncogenic MYC(N) expression, but not in the less common mesenchymal neuroblastoma lines (Fig. 5d and Extended Data Fig. 3c). Pharmacologically inhibiting CTH using propargylglycine (PPG)^33^ sensitized adrenergic, but not mesenchymal, high *MYCN* cell lines to either erastin- or imidazole ketone erastin (IKE)-induced cell death (Fig. 5e). Knockdown of AHCY in adrenergic high *MYCN* but not mesenchymal neuroblastoma cells impaired colony formation, which was associated with reduced GSH levels and reduction of GSH reduced-to-oxidized ratios (Fig. 5f, g). In summary, these data indicate that transsulfuration provides an internal cysteine source for GSH biosynthesis protecting high *MYCN* adrenergic neuroblastoma cells from ferroptosis.

Intracellular cysteine is required for two rate-limiting cellular processes: GSH-mediated ROS clearance and production of building blocks in the synthesis of proteins, nucleotides and lipids^34^ (Fig. 5a). In line with this, protein synthesis inhibition with cycloheximide increased cysteine and total GSH levels (Extended Data Fig. 3d). Notably, reduction/deprivation of cystine (Cys_2_) in the medium drastically reduced intracellular cysteine and GSH in the high *MYCN* state before ferroptosis (Fig. 2a,b) but did not affect cell cycle progression and only moderately reduced protein synthesis (Extended Data Fig. 3e,f). MYCN protein levels were not affected on cystine deprivation compared to methionine- or Gln-deprived cells (Extended Data Fig. 3g). These data suggest robust cysteine channeling into protein synthesis under limited cystine supply when GSH production is already diminished.

To investigate this further, we performed transcriptomic analysis of IMR5/75 cells cultured in cystine-free medium or inhibiting the x_c_^−^ system with erastin. This revealed activation of a stress response^35^ before ferroptosis that channeled cysteine into protein synthesis by inducing *CARS*^31^ and cysteine recycling from GSH (Fig. 2d and Extended Data Fig. 3h–j). *HMOX1* was also activated in these cells, suggesting that the free iron pool is increased before inducing ferroptosis^36^ (Fig. 2d and Extended Data Fig. 3h–j). Inhibiting CARS prevented ferroptosis in the cystine-deprived high *MYCN* state, highlighting the competition for intracellularly synthesized cysteine between protein synthesis and redox balance (Fig. 5h). Taken together, transsulfuration supplies cysteine for both protein and GSH synthesis in adrenergic *MYCN*-amplified neuroblastoma cells but prioritizes cysteine for protein synthesis at the expense of GSH and redox balance when cystine uptake is limited, thus triggering ferroptosis.

**Extended Data Fig. 4:**
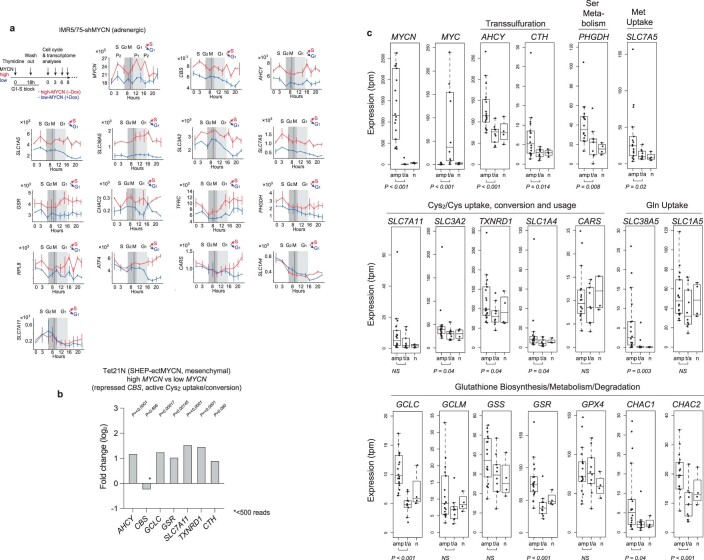
Expression profiles of neuroblastoma cell lines. **a**, Time-resolved gene expression profiles during cell cycle progression and cell cycle analysis distinguish pervasive MYCN functions from indirect effects related to cell proliferation rate in high *MYCN* and low *MYCN* IMR5/75 cells: *MYCN* gene expression profile reveals two cell cycle-related peaks (P1 before G1-S transition and P2 before S-G2/M). **b**, Fold change (log_2_) of gene expression in mesenchymal Tet21N (SH-EP-ectMYCN) cells harboring an inducible *MYCN* transgene upon MYCN induction. Tet21N and parental SH-EP cells have low *CBS* expression and inactive transsulfuration. MYCN induction has a significant effect on *AHCY*, *GCLC, GSR, SLC7A11* and *TXNRD1* but not on *CBS* expression; experiment replicated 3 times. **c**, Expression of transsulfuration, amino acid uptake/metabolism and glutathione biosynthesis/metabolism/degradation genes in 32 neuroblastoma cell lines (RNA-seq): *MYCN*-amplified (amp) vs. *MYCN*/*MYC*-translocated/activated (t/a) cell lines, MYC(N)-non-expressors (n) (Wilcoxon rank-sum test; NS = not significant). Center line indicates the median value, lower and upper hinges represent the 25th and 75th percentiles, respectively, and whiskers denote 1.5× interquartile range. Each dot corresponds to one sample; *p*-values as indicated. Source data

**Extended Data Fig. 5:**
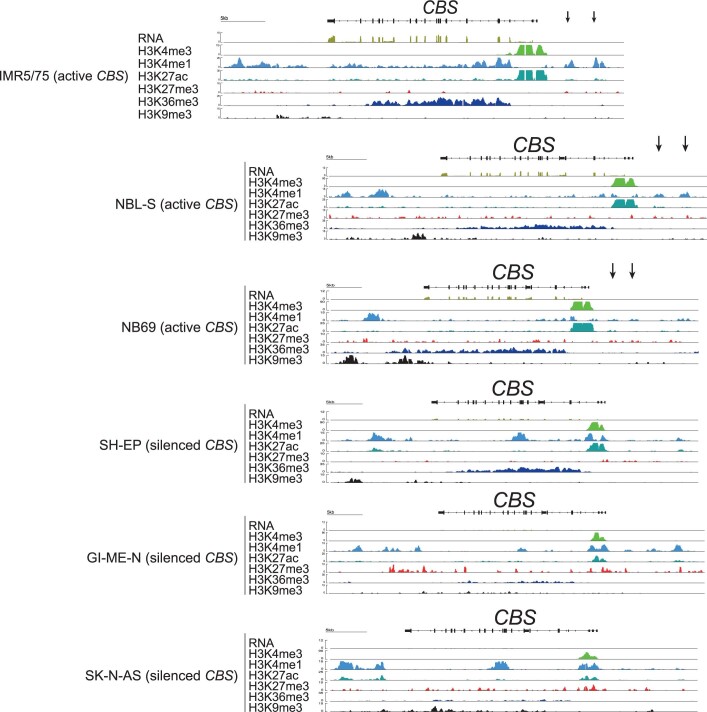
ChIP-seq data for histone modifications. RNA-seq normalized reads and input normalized read counts of ChIP-seq experiments for histone modifications at the *CBS* gene locus in adrenergic *MYCN*-amplified IMR5/75, *MYCN*-translocated NBL-S, and *MYC*-translocated NB69 cells with high *CBS* expression and active transsulfuration, mesenchymal *MYCN*-normal diploid SH-EP, GI-M-EN, and SK-N-AS cells with elevated MYC expression due to translocations or unknown mechanism (GI-M-EN) and very low/absent *CBS* expression and inactive transsulfuration. Source data

**Extended Data Fig. 6:**
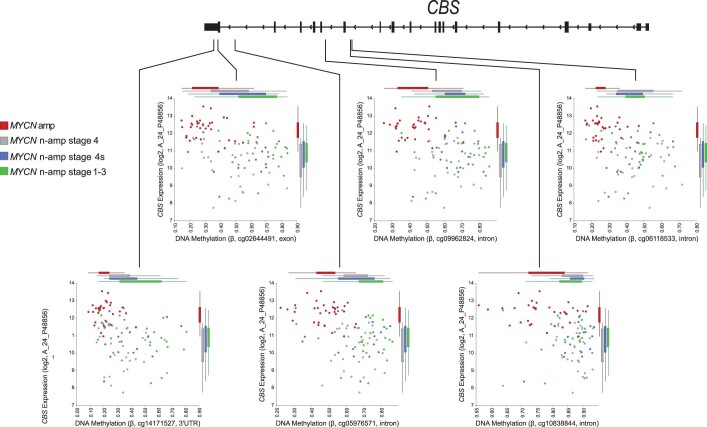
Methylation profiles and CBS expression in tumor samples. Genomic position of *CBS*-annotated CpGs whose methylation is significantly associated with *CBS* expression and patient risk (p < 0.01, Wilcoxon rank-sum statistics and p < 0.05, Fisher exact test). DNA methylation assessed by Infinium Human Methylation 450 BeadChips, and *CBS* expression assessed by 44k customized Agilent oligonucleotide microarrays in 105 primary neuroblastomas (GEO accession GSE73518^18^). For all *CBS* CpGs whose methylation significantly correlated with *CBS* expression and patient risk, hypomethylation was associated with *CBS* upregulation and high-risk disease. R2 Genomics Analysis and Visualization Platform (http://r2.amc.nl) was used for data visualization. Source data

Next, we asked how cysteine metabolism and redox homeostasis are affected by oncogenic MYC(N) activity in the adrenergic (active transsulfuration) or mesenchymal (inactive transsulfuration) neuroblastoma subtypes^8^. Adrenergic cells upregulated three key enzymes in transsulfuration, *CBS*, *AHCY* and D-3-phosphoglycerate dehydrogenase (*PHGDH*), in the high *MYCN* state, while the x_c_^−^ system (*SLC7A11*) controlling cystine uptake was unaffected by changes in *MYCN* (Extended Data Fig. 4a)^37^. In a panel of 32 neuroblastoma cell lines, *MYCN* or *MYC* amplification or translocation in adrenergic subtypes was accompanied by upregulated *CBS* expression, while *MYC* translocation/activation in mesenchymal cell lines was not (Fig. 5i). Ectopically expressing *MYCN* in mesenchymal Tet21N cells left transsulfuration via its rate-limiting enzyme *CBS* unchanged but induced *SLC7A11*, *GSR*, *GCLC* and thioredoxin reductase 1 (*TXNRD1*), the last one being significantly expressed in *MYCN*-amplified primary neuroblastomas (Extended Data Fig. 4b,c)^37^. At the gene regulatory level, we found that the *CBS* locus harbored both activating (H3K27ac, H3K4me3) and silencing (H3K27me3) histone modifications, with the former being increased and the latter decreased in the presence of amplified *MYCN* in adrenergic cells compared to non-amplified or mesenchymal cells (Fig. 5j and Extended Data Fig. 5). In primary neuroblastomas, differences in *CBS* expression correlated with histone modifications and methylation of intragenic CpGs dependent on genomic *MYCN* status (Fig. 5k and Extended Data Fig. 6). Together, this suggests that transsulfuration is active in the adrenergic state and regulated at the epigenetic level in high *MYCN* cells.

**Fig. 6:**
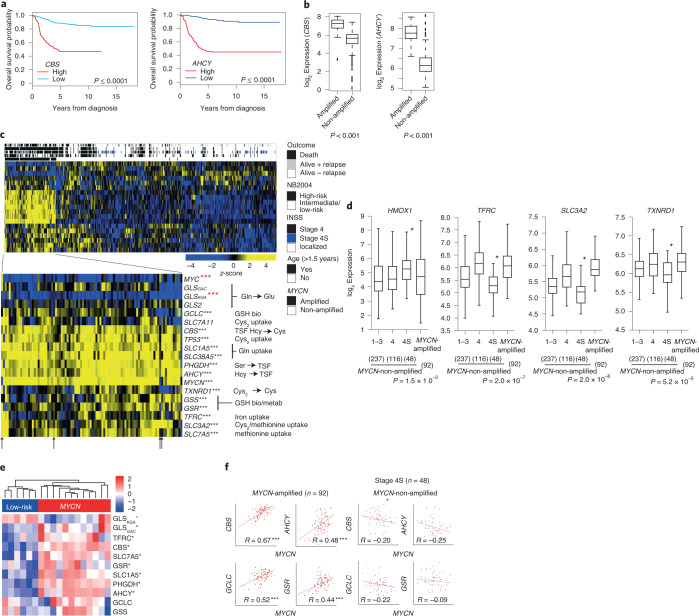
Gene and protein markers indicating ferroptosis sensitivity in high- and low-risk neuroblastomas. **a**, Kaplan–Meier overall survival estimates for subgroups defined by *CBS* expression, high *CBS* (*n* = 123) and low *CBS* (*n* = 375). Kaplan–Meier overall survival estimates for subgroups defined by *AHCY* expression. The cutoff values for the dichotomization of *AHCY* expression were estimated by maximally selected log-rank statistics, high *AHCY* expression (*n* = 165) and low *AHCY* expression (*n* = 333). **b**, *CBS* and *AHCY* expression in *MYCN* status-dependent expression in 498 primary neuroblastomas (92 *MYCN*-amplified, 406 *MYCN* non-amplified tumors, RNA-seq); Wilcoxon rank-sum test. **c**, Gene expression heatmap of subgroups hierarchically clustered with average linkage and non-centralized correlation distance function. Row: transcript; column: sample, according the NB2004 study. ***P < 0.001 (black) higher and ***P < 0.001 (red) lower gene expression. Wilcoxon rank-sum test; the arrows mark *MYCN*-non-amplified tumors. **d**, Differential expression of cysteine uptake and iron-regulating genes in primary neuroblastomas dependent on the stage of the disease and *MYCN* amplification status. Differential expression in stage 4S versus all other neuroblastoma subtypes tested by Wilcoxon rank-sum test. **e**, Differential expression of proteins mediating GSH synthesis/metabolism and GSH-relevant amino acid uptake/metabolism compared between *MYCN*-amplified and low-risk, normal *MYCN* tumors. *P ≤ 0.01; higher (black) and lower (red) protein expression. *P* values were calculated using a two-sided Welch’s *t*-test. Exact *P* values are given in the source data. Intensities are given as *z*-scores. See the legend for color-coding. **f**, Pearson’s correlation analysis of *MYCN* expression and GSH synthesis/metabolism and transsulfuration genes in *MYCN*-amplified (*n* = 92) and *MYCN*-non-amplified, low-risk stage 4S (*n* = 48) tumors. Box plots: the center line indicates the median value, the lower and upper hinges represent the 25th and 75th percentiles, respectively and the whiskers denote the 1.5× the IQR. Each dot corresponds to one sample; Wilcoxon rank-sum test; *P* values as indicated. Source data

We found that the levels of *CBS* and *AHCY* were associated with poor patient survival (Fig. 6a). Global gene expression profiles from 498 primary neuroblastomas^38^ confirmed elevated *AHCY* and *CBS* in *MYCN*-amplified neuroblastomas (Fig. 6b,c). Higher *HMOX1* and lower *SLC3A2* and *TXNRD1* expression were found in stage 4S tumors (Fig. 6d), which tend to regress spontaneously. CBS, AHCY and PHGDH expression was also elevated in mass spectrometry-based global proteomes from *MYCN*-amplified neuroblastoma tumors (Fig. 6e). *SLC7A11* antiporter expression did not correlate with *MYCN* amplification or other risk factors for poor patient outcomes (Fig. 6c). In addition, genes involved in Gln (that is, *SLC38A5*, *SLC1A5*), methionine (that is, *SLC7A5*) and iron uptake (*TFRC*) had higher expression in *MYCN*-amplified or *MYCN*-overexpressed neuroblastomas; *TFRC* was recently associated with ferroptosis in neuroblastomas^12^ (Fig. 6c,d and Extended Data Fig. 4c). Genes associated with GSH synthesis/metabolism (that is, *GCLC*, *GSR*) were also upregulated both at the transcript and protein level (Fig. 6c,e,f). Higher expression of the mitochondrial glutaminase (*GLS*_*GAC*_)^24,39^ and lower expression of the cytoplasmatic glutaminase (*GLS*_*KGA*_) isoforms were observed in *MYCN*-amplified tumor transcriptomes and proteomes (Fig. 6c,e), suggesting dependence on mitochondrial glutaminolysis in these neuroblastomas. Together, our results show that *MYCN*-amplified neuroblastomas increase transsulfuration activity, iron import, glutaminolysis and GSH production through coordinated changes in gene expression thereby increasing the susceptibility to ferroptosis.

### Cyst(e)ine and GPX4 inhibition as therapeutic targets

**Fig. 7:**
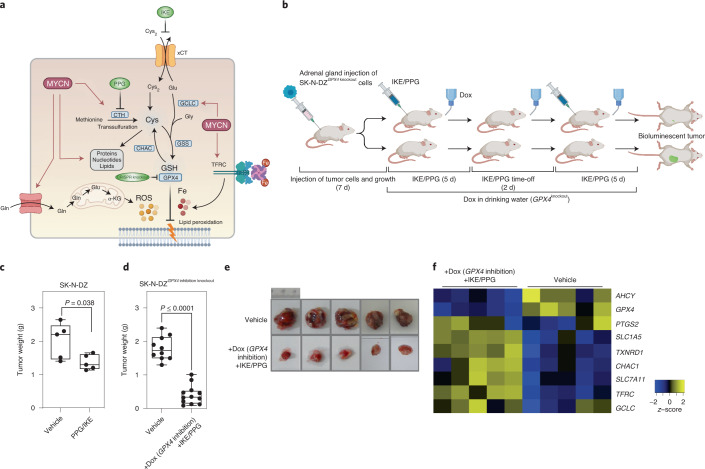
Triple combination inhibiting GPX4, cystine uptake and transsulfuration eliminates *MYCN*-amplified tumors in vivo. **a**, Regulation of pro- and anti-ferroptotic players by oncogenic MYCN in *MYCN*-amplified neuroblastoma cells that may trigger ferroptosis when both cysteine and GSH availability is limited. Therapeutic intervention points are indicated in green. **b**, Dosage scheme applied with either double (IKE/PPG) or triple combination (*GPX4* knockout, IKE and PPG). **c**,**d**, Tumor weight after combination treatment with IKE and PPG (*n* = 5 mice per group; **c**) and the triple combination of GPX4 reduction plus IKE/PPG treatment (*n* = 9 in the control group and *n* = 12 mice in the treated group; **d**). **e**, Representative photographs of fully grown tumors in the vehicle group versus residual tumors after triple combination. **f**, Transcriptional changes of ferroptosis markers after GPX4 reduction plus IKE/PPG in residual tumor tissue (*n* = 5 tumor samples from each group). Statistical analysis was performed using a one-tailed Student’s *t*-test for the in vivo experiments. Box plots: the center line indicates the median value and the lower and upper hinges represent the minimum and maximum points. Each dot corresponds to one sample; *P* values as indicated. Panels **a** and **b** created with BioRender. Source data

To exploit our findings in vivo, we first tested simultaneous inhibition of cysteine uptake and transsulfuration in vitro using IKE, an erastin analog with acceptable pharmacokinetic properties^40,41^, and PPG^33^ (Fig. 7a). We observed a strong synergistic effect with the two drugs in only *MYCN*-amplified adrenergic cells (Fig. 5e). We then injected mice orthotopically with SK-N-DZ neuroblastoma cells and treated them for two 5-d cycles with a combination of IKE 45 mg kg d^−1^ and PPG 45 mg kg d^−1^ (Fig. 7b). When combining these two drugs, we observed a 60% reduction in tumor growth in *MYCN*-amplified SK-N-DZ-driven tumors (Fig. 7c). These data suggest that *MYCN*-amplified neuroblastoma cells with very high levels of MYCN are sensitive to a reduction of the intracellular cysteine pool by simultaneous inhibition of cystine import and cysteine synthesis via transsulfuration.

Next, we combined this protocol for reducing cellular cysteine with genetic targeting of GPX4 activity (Fig. 7b). We observed a robust effect with complete remission in most animals (Fig. 7d,e). Transcriptional profiling of residual small tumors revealed induction of ferroptosis markers after combined inhibition of cystine uptake/cysteine synthesis and GPX4 compared to tumors treated with vehicle control (Fig. 7f). These data provide strong in vivo evidence that concomitant reduction of cystine uptake, transsulfuration and GPX4 activity can be utilized as a new therapeutic strategy for high-risk, *MYCN*-amplified neuroblastomas.

## Discussion

We demonstrated that oncogenic *MYCN* sensitizes neuroblastoma cells to ferroptosis when intracellular cysteine availability for GSH synthesis and the cystine/cysteine redox cycle are limited. A high *MYCN* state in neuroblastoma cells sensitizes them to lipid peroxidation, which in combination with acute intracellular cysteine reduction triggers massive ferroptotic cell death. Our study shows transsulfuration and GSH redox activity to be crucial to escape ferroptosis, whereas Gln import and glutaminolysis are required for ferroptosis in neuroblastoma cells with oncogenic *MYCN* (Fig. 7a). Expression levels of key genes for these processes are correlated with *MYCN* expression in high-risk tumors. The recently identified adrenergic and mesenchymal subtypes^8^ also appear to determine how cysteine is maintained in neuroblastoma cells, with transsulfuration only being activated by oncogenic MYC(N) in adrenergic cells, where *CBS* is accessible and not epigenetically repressed as in mesenchymal cells.

In this study, we describe metabolic rewiring in *MYCN*-amplified adrenergic neuroblastoma cells, where high consumption of cysteine used for the synthesis of cellular building blocks at the expense of GSH synthesis and ROS clearance creates a new *MYCN*-dependent liability. To prove that this liability can be exploited as a new therapeutic concept, we established and optimized an orthotopic model for neuroblastoma using intra-adrenal gland tumor cell transplantation. This model allowed robust in vivo testing of ferroptosis induction: we obtained remarkable tumor remission in high *MYCN* neuroblastoma by combining inhibition of (1) cystine import using IKE and (2) transsulfuration using PPG together with (3) CRISPR–Cas9-mediated *GPX4* deletion. Among the different parameters tested, this was the most effective strategy revealing almost complete tumor remission. Currently there are no GPX4 inhibitors for in vivo use, hence future improvements of this therapeutic strategy would involve the development of potent GPX4 inhibitors with optimal pharmacokinetics and pharmacodynamics.

Recent studies^11,12^ highlighted the role of iron in *MYCN*-dependent neuroblastoma and ferroptosis. The *TFRC* gene, involved in iron uptake, is activated by *MYC* in several cell types enhancing cellular proliferation^42^. In line with these studies, we showed a synergistic effect of iron in drug-induced ferroptosis in *MYCN*-amplified cells. In addition, we demonstrated that the iron chelator CPX rescues ferroptotic cell death under cystine deprivation conditions (Extended Data Fig. 1h). We further observed high expression of *TFRC* in *MYCN*-amplified cell lines and tumors (Fig. 6c and Extended Data Figs. 3j and 4a) reflected on the protein level (Fig. 6e). Similarly, we observed *HMOX1* upregulation, involved in increasing the labile iron pool, in cystine-deprived neuroblastoma cells before ferroptosis induction. Together, this highlights a prominent role for iron metabolism in promoting ferroptosis in neuroblastoma cells.

We have described a detailed mechanism that explains how cystine deprivation triggers ferroptosis in *MYCN*-amplified tumors, which depends on the (epi)genetic context of adrenergic or mesenchymal subtypes, the later having transsulfuration silenced. These results differ from Floros et al.^11^, who suggested that high *MYCN* mediates upregulation of *SLC7A11* in neuroblastoma cells. In contrast, our results indicate that in most, if not all, adrenergic neuroblastoma cells (>95% of neuroblastomas) high *MYCN* fails to boost cystine uptake via x_c_^−^. Instead, MYCN strongly activates transsulfuration, the intracellular conversion of methionine to cysteine. In general, neuroblastoma cells have very low *SLC7A11* expression compared to other cancer types, which may explain the remarkable sensitivity of neuroblastoma cells to ferroptosis stimuli. Our results demonstrate that the uptake of cystine via x_c_^−^ is not regulated on tumor progression in adrenergic *MYCN*-amplified cells. Upregulation of *SLC7A11* by MYC oncoproteins was only found in cells of the rare mesenchymal subtype, having epigenetically silenced transsulfuration. Therefore, upregulation of *SLC7A11* may become relevant in the relapse scenario, which is associated with elevated proportions of mesenchymal cells.

Intriguingly, our data may also explain spontaneous neuroblastoma regression, the mechanisms of which have remained elusive. In low-risk, metastatic neuroblastomas (stage 4S), which are likely to regress spontaneously^5^, we identified multiple markers predisposing them to ferroptosis (Fig. 7a), including: (1) failure to upregulate enzymes involved in GSH biosynthesis (GCLC) and redox activity (GSR) and transsulfuration (AHCY, CBS) with increasing MYCN activity (Fig. 6f); (2) *CBS* silencing by intragenic CpG methylation (Extended Data Fig. 6a); (3) *HMOX1* upregulation to increase free iron pools; and (4) *SLC3A2* and *TXNRD1* downregulation (Fig. 6d). Spontaneous regression may be the physiological resolution of this cellular state sensitive to ferroptosis. Unlike low-risk tumors, *MYCN*-amplified neuroblastomas appear to metabolically adapt to survive events that deplete intracellular cysteine, such as high systemic glutamate present in the first two years of life^43^. The cysteine requirement of cancers dependent on oncogenic *MYCN* activity creates a previously unknown Achilles’ heel that could be exploited to selectively induce ferroptosis for treatment. Our findings identify cysteine uptake, transsulfuration and the lipid peroxidation-specific scavenging system as vulnerabilities in cancer cells driven by oncogenic MYC(N) activity such as *MYCN*-amplified neuroblastomas.

## Methods

### Ethics

All patients with neuroblastoma were enrolled in the German Neuroblastoma Trial (NB97, NB2004, NB 2016) and approved by the ethics committee of the University of Cologne. All studies involving mice and experimental protocols were conducted in compliance with German Cancer Research Center guidelines and approved by the governmental review board of the state of Baden-Württemberg.

### Experimental in vitro procedures

#### Cell culture

Human neuroblastoma cells (IMR5/75, KELLY, SiMa, NBL-S, SK-N-FI, SH-SY5Y, NB69, SK-N-DZ, SH-EP, GI-ME-N) were cultivated at 37 °C with 5% CO_2_ in Roswell Park Memorial Institute (RPMI) 1640 medium (Gibco) supplemented with 10% fetal calf dialyzed serum (Gibco) and penicillin/streptomycin (AppliChem). The KELLY and SiMa cell lines were purchased from Deutsche Sammlung von Mikroorganismen und Zellkulturen. SK-N-FI cells were purchased from ATCC. NBL-S and Tet21N (SH-EP) cells were provided by G.M. Brodeur and W. Lutz, respectively. Tunable cell lines, IMR5/75 *MYCN* short hairpin RNA (shRNA) and SH-EP *MYCN* transgene (Tet21N) cells were generated and cultured as described previously^15,17^. Cell line identity/unique single-nucleotide polymorphism profiles were confirmed by the Multiplexion Multiplex Cell Authentication service as described recently^44^. The purity of cell lines was validated using the Multiplex Cell Contamination Test (Multiplexion) as described recently^44^. No *Mycoplasma*, squirrel monkey retrovirus or interspecies contamination was detected. To assess the effects of amino acid deprivation, cells were cultivated using modified amino acid-free DMEM powder (PAN-Biotech) supplemented with individual amino acids (Sigma-Aldrich) as indicated, at final concentrations used in standard DMEM. For the 3D culture experiments, we used the hanging drop method. Twenty thousand cells were placed in hanging drop culture and incubated under physiological conditions until they form 3D spheroids.

#### Analysis of cell viability and proliferation

The impact of various treatments on cellular proliferation/viability was assessed using a sulforhodamine B (SRB)^45^ or CellTiter-Blue (Promega Corporation) cell assay. To determine changes in cellular proliferation, approximately 2 × 10^4^ cells were seeded per well (48-well format for the SRB assay and 96-well format for the CellTiter-Blue assay) in full medium. After 24 h, cells were washed with PBS, fed with the chosen medium and treated as indicated. Cell viability was analyzed in full or Cys_2_-free medium cotreated with 10058-F4 (30 µM, catalog no. F3680; Sigma-Aldrich), Z-VAD-FMK (30 µM; catalog no. sc-3067; Santa Cruz Biotechnology), bafilomycin A1 (30 nM, catalog no. sc-201550; Santa Cruz Biotechnology), necrostatin-1 (2 µM, catalog no. N9037; Sigma-Aldrich), Fer-1 (5 µM, catalog no. SML0583; Sigma-Aldrich), Trolox (100 µM, catalog no. 238813; Sigma-Aldrich), aminooxyacetate (500 µM, Sigma-Aldrich), dimethyloxalylglycine, N-(methoxyoxoacetyl)-glycine methyl ester (5 mM, Sigma-Aldrich), CPX (1 µM, catalog no. sc-204688; Santa Cruz Biotechnology), D9 (synthesized and provided by Bayer Pharma AG), erastin (Cay17754; Biomol), IKE (MedChemExpress), 2-mercaptoethanol (50 µM; Sigma-Aldrich), glutaminase inhibitors BPTES (catalog no. SML0601; Sigma-Aldrich) and compound 968 (catalog no. 352010; Merck Millipore), RSL3 (MedchemExpress), sulfasalazine (catalog no. 599-79-1; Sigma-Aldrich), PPG (1 mM, catalog no. P7888; Sigma-Aldrich). DNA content analysis was performed by fixing cells with 4% paraformaldehyde and staining with FxCycle Violet Stain (Thermo Fisher Scientific) followed by fluorescence-activated cell sorting (FACS) using a MACSQuant Flow Cytometer (Miltenyi Biotec). To assess the effects of Cys_2_ deprivation on cell viability, cells were washed and fed with Cys_2_-free DMEM supplemented with 10% dialyzed FCS, 200 µM L-methionine (catalog no. 63-68-3; Sigma-Aldrich) and 4 mM Gln (catalog no. 25030081; Gibco) 48 h after seeding. To determine the cell death rescue potential, Cys_2_-deprived cells were also cotreated with Hcy (catalog no. 69453; Sigma-Aldrich), Cysta (catalog no. C7505; Sigma-Aldrich) or GSH (2 mM, catalog no. G4251; Sigma-Aldrich). We further compared effects on cell viability after Cys_2_ and Gln depletion or Gln excess using the following conditions: full RPMI (208 µM Cys_2_, 2.055 mM Gln, 0.136 µM Glu); low Cys_2_ (5 µM); low Gln (0.2 mM); and high Gln (2.5 mM). Fluorescence was read (540/580 nm) 24 h and 48 h after deprivation/treatment. Doubling times were calculated using two different methods: (1) impedance measurements were assessed using the RTCA system (Roche) by seeding at different cell densities and registering impedance signals every 20 min; (2) standard growth curves were generated by counting cells by FACS at the indicated time points and excluding propidium iodide-positive cells as necrotic. In both methods, exponential curves were fitted and doubling times calculated.

#### Inducible stable *AHCY* knockdown cell cultures

Stable IMR5/75 and SH-EP cell lines expressing shRNA against AHCY under control of the Tet repressor were generated stepwise as described previously^15^ using the following oligonucleotide sequence to target *AHCY*: forward: gatccccGGATCACTACCGCTACTGAttcaagagaTCAGTAGCGGTAGTGATCCtttttggaaa; reverse: gcttttccaaaaaGGATCACTACCGCTACTGAtctcttgaaTCAGTAGCGGTAGTGATCCggg.

IMR5/75 (5,000 cells per well) and SH-EP-AHCYsh (1,000 cells per well) were seeded in 6-well plates and simultaneously treated with Dox (1 µg ml^−1^) to induce the *AHCY*-targeting shRNA. Cells were fixed (11% glutaraldehyde; Sigma-Aldrich) and Giemsa-stained five (SH-EP-AHCYsh) or 7 d (IMR5/75-AHCYsh) later. Colony counting was performed using a GelDoc Documentation System and Quantity One software version 4.6.6 (Bio-Rad Laboratories) and quantification using Microsoft Excel 2016.

#### Quantification of amino acids

Pellets of 2 × 10^6^ cells were extracted with 0.1 ml ice-cold 0.1 M HCl. Non-thiol-containing amino acids were quantified after specific fluorescent labeling with AccQ-TagTM (Waters) as described previously^46^. Cysteine and GSH levels were determined after labeling with monobromobimane (Calbiochem) as described previously^47^.

#### Western blot analysis of proteins in cell extracts

Whole cell lysates were prepared and protein expression was visualized as described previously^48^. Protein lysates (20 μg per lane) were separated on 12.5% SDS–polyacrylamide gel electrophoresis. Blots were probed with antibodies directed against MYCN (1:1,000 dilution, catalog no. sc-53993; Santa Cruz Biotechnology), c-MYC (1:1,000 dilution, catalog no. ab32072; Abcam), CTH (1:1,000 dilution, catalog no. ab54573; Abcam), SAHH (A-11) (AHCY antibody) (1:1,000 dilution, catalog no. sc-271389; Santa Cruz Biotechnology), GPX4 (1:1,000 dilution, catalog no. ab41787; Abcam), CARS (1:1,000 dilution, catalog no. ab126714; Abcam), glutaminase 1 (1:40,000 dilution, catalog no. ab156876; Abcam), vinculin (1:1,000 dilution, catalog no. sc-73614; Santa Cruz Biotechnology) or horseradish peroxidase-conjugated anti-β-actin (1:5,000, catalog no. ab20272; Abcam). Peroxidase-AffiniPure goat anti-mouse IgG (H+L) (1:1,000 dilution, catalog no. 115-035-003; Dianova) or peroxidase AffiniPure goat anti-rabbit IgG (H+L) (1:1,000 dilution, catalog no. 111-035-144; Dianova) antibodies were used as secondary antibodies. Proteins were visualized using enhanced chemiluminescence detection reagents (GE Healthcare) and a chemiluminescence reader (VILBER). Protein quantification was performed with ImageJ (https://imagej.net).

### Flow cytometry

#### Analysis of intracellular ROS levels and lipid peroxidation

**Extended Data Fig. 7:**
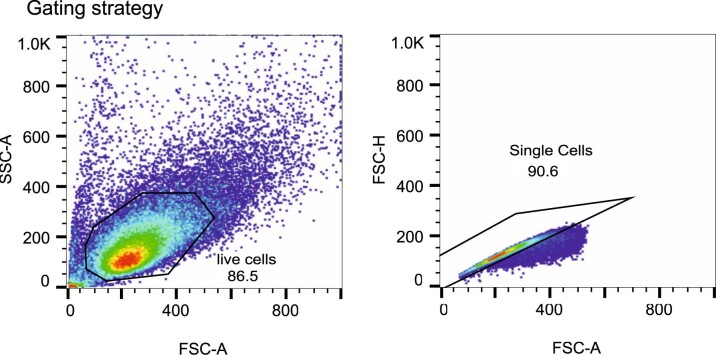
Gating strategy for flow cytometry data. For all samples, an initial manual gate in SSC-A by FSC-A was set to identify live cells and exclude debris. From the live cells, a rectangular gate was set on FSC-H by FSC-A to exclude doublets, meaning cells off the diagonal. If DNA staining was carried out, an additional gate was set on DNA (Violet channel)-W by DNA-A to exclude additional cell debris or DNA doublets.

Low *MYCN* populations were established by incubating cells with 1 µg ml^−1^ Dox at least 48 h before further treatment. Cells were then fed either with full or cystine-free medium and cotreated with Fer-1 (5 µM), liproxstatin-1 (Lip-1) (1 µM; catalog no. SML1414; Sigma-Aldrich), CPX (1 µM), Trolox (100 µM) or GSH (2 mM, catalog no. G4251; Sigma-Aldrich) for 20 h. Lipid peroxidation was analyzed with the C11-BODIPY BD FACSAria III cell sorter. Total intracellular ROS levels were determined using CellROX (Thermo Fisher Scientific). Gating strategy is shown in Extended Data Fig. 7.

### MYCN synthetic lethal screen

#### Large-scale druggable genome siRNA screen

For high-throughput screening, a Silencer Select siRNA custom library (catalog no. 4404034; Ambion) was used encompassing 31,242 unpooled siRNAs targeting 10,414 genes (3 siRNAs per gene). Lipofectamine RNAiMax Transfection Reagent (Thermo Fisher Scientific) only and ON-TARGETplus Non-targeting siRNA no. 1 (Dharmacon) served as negative transfection controls; PLK1 (Silencer Select siRNA no. 1; Ambion) served as positive control. Liquid reverse transfection was performed in 384-well plates (2,100 cells per well) using a Freedom EVO 200 robotic platform. Two treatment conditions were screened in triplicate: (1) culture medium only (IMR5/75 high *MYCN*); and (2) plus Dox (1 µg ml^−1^ final concentration) to induce the shRNA targeting *MYCN* (IMR5/75 low *MYCN*); 96 h after transfection, cells were fixed with 11% glutaraldehyde and subsequently Hoechst-stained (10 mg ml^−1^ stock in 1 × PBS, 1:2,500; Invitrogen). The number of Hoechst-positive cell nuclei was determined using an OPERA fluorescence microscope based on nine sites per well and a BHC in-house program. We applied redundant siRNA activity (RSA) to the ratio between high and low *MYCN* data to select top-ranked hits and a false discovery rate (FDR) of 0.2.

#### Transient siRNA-mediated gene knockdown

IMR5/75 cells were seeded in 96-well plates (3,000 cells per well); 24 h later, they were transiently transfected with a mix of RNAiMax (0.04 µl per well; Thermo Fisher Scientific) and 0.01 µM per well of siRNA according to the manufacturer’s instructions. siRNA sequences are listed in Supplementary Table 2.

### Generation of inducible Cas9 neuroblastoma cell lines

#### Molecular cloning

The CRISPR–Cas9-mediated inducible knockout experiments were performed using the lentiviral pCW-Cas9-EGFP plasmid. The plasmid was obtained as follows: the lentiviral pCW-Cas9 plasmid (plasmid no. 50661; Addgene) was subcloned by cutting it with the restriction enzymes HincII and XbaI (New England Biolabs). Next, a gBlock DNA fragment (Integrated DNA Technologies) encoding enhanced green fluorescent protein (eGFP) with complementary overhangs was cloned into the plasmid backbone thus replacing the puromycin resistance gene and generating the pCW-Cas9-EGFP plasmid. Guide RNAs (gRNAs) for the CRISPR knockout experiments were designed with Benchling (https://www.benchling.com) using the human reference genome GRCh38. Oligonucleotides were ordered (Sigma-Aldrich) with complementary overhangs to the lentiviral delivery plasmid backbone pLKO5.sgRNA.EFS.tRFP6572 and listed in Supplementary Table 2.

#### Lentivirus production

Lentivirus production was performed using a second-generation lentiviral system and a Calcium Phosphate Transfection Kit (Invitrogen) in HEK 293T cells. Briefly, early passaged HEK 293T cells were cotransfected with the lentiviral transfer plasmid, a packaging plasmid (psPAX2; plasmid no. 12260; Addgene), and with a plasmid coding for the VSV-G envelope (pMD2.G; plasmid no. 12259; Addgene). All experimental procedures for lentivirus production were performed in a biosafety level 2 laboratory.

The SK-N-DZ cell line was previously transduced with lentiviral particles carrying the luciferase reporter pLX-puroR-Luc at a multiplicity of infection (MOI) of 0.3. After recovery, resistant cells were transduced with pCW-Cas9-EGFP lentiviral particles at an MOI of 0.3. Polyclonal cell lines were maintained in DMEM supplemented with 10% tetracycline-free FCS (Clontech Laboratories). Next, cells expressing eGFP and the luciferase reporter were transduced with lentiviral particles carrying the gRNA targeting *GPX4* at an MOI of 0.3. To generate monoclonal CRISPR cell lines, cells were individualized based on eGFP and RFP657 expression using FACS. In vitro Cas9 expression was induced supplementing the culture medium with 1 µg ml^−1^ Dox (Sigma-Aldrich).

#### In vivo orthotopic mouse experiments

All studies involving mice and experimental protocols were conducted in compliance with German Cancer Research Center guidelines and approved by the governmental review board of the state of Baden-Württemberg, Karlsruhe District Council, under authorization no. G-176/19, according to German legal regulations. The mouse strains used in the study were NOD.Cg-Prkdc^scid^Il2rgtm1^Wjl^/SzJ (stock no. 005557; The Jackson Laboratory). Female mice (3–4 months old) were used for the experiments. Mice were housed in individually ventilated cages under temperature and humidity control. Cages contained an enriched environment with bedding material. To generate orthotopic mouse models for neuroblastoma, 2 × 10^5^ SK-N-DZ cells were transplanted into the right adrenal gland after the surgical site was prepared^49^. Cells were resuspended in a 1:1 (vol/vol) mix of growth factor-reduced Matrigel (Corning) and PBS. Overall, 20 µl of this cell suspension was injected into the right adrenal gland of anesthetized mice. After tumor cell transplantation, we monitored mice for evidence of tumor development by bioluminescent signal using an IVIS Spectrum Xenogen device (Caliper Life Sciences). We observed a clear signal from the tumors 1 week after the injection of 2 × 10^5^ SK-N-DZ cells. IKE and PPG were used at a concentration of 45 mg kg d^−1^ through intraperitoneal injection. Animal health was monitored daily and mice were euthanized as soon as they reached the termination criteria defined in the procedure. Sample size was calculated with the help of a biostatistician using R v.3.4.0. Assumptions for the power analysis were as follows: Alpha error, 5%; Beta error, 20%. Mice were randomized into treatment groups before treatment. In case animals had to be euthanized before the predefined end point (due to weight loss or other termination criteria), they were excluded from any downstream analyses. All animal experiments (apart from animal treatment) were blinded during the experiments and follow-up assessment.

### Transcript profiling of tumors and cell lines

#### Microarray analysis

RNA isolated from the remaining tumors (five vehicle-treated versus five triple combination of *GPX4* knockout + IKE + PPG) was analyzed using the Affymetrix GeneChip Clariom S Assay. Microarray data (GSE192976) were processed using a modification of a pipeline described previously^50^. Briefly, raw CEL files were robust multichip average-normalized and two-group comparisons were performed using the limma package v.3.46 (Bioconductor) with an empirical Bayes test for differential expression. An FDR < 0.1 was regarded as statistically significant.

#### RNA sequencing

Total RNA was isolated using the miRNeasy Mini Kit (QIAGEN) according to the manufacturer’s protocols. RNA libraries were prepared using the NEBNext Ultra Directional RNA Library Prep Kit for Illumina (New England Biolabs). Raw RNA sequencing (RNA-seq) sequences of neuroblastoma cell lines and model systems were mapped to the UCSC hg19 genome using STAR^51^ v.2.5.3a with default parameters. RNA-seq sequences from the IMR5/75 high and low *MYCN* cells were processed as described previously^37^. Differentially expressed genes were identified using the edgeR v.3.20.9 generalized linear model (glmFit) approach in R v3.4.3. The expression profiles of the high *MYCN* depletion/rescue experiments were visualized in R v4.3.0 using the gplots v.3.1.1 function heatmap.2, after filtering out lowly expressed genes (count per million ≤ 1) and normalizing for library size using trimmed mean of *M*-values normalization as implemented in edgeR^52^ v.3.20.9. RNA-seq expression profiles from 498 primary neuroblastomas^38^ (GSE49711) were analyzed. The Wilcoxon rank-sum test was used to test the association between candidate gene expression and amplified *MYCN* oncogene. Maximally selected log-rank statistics were used to describe the relationship between patient survival and candidate gene expression; the resulting expression cutoff points were used for dichotomization. Survival curves were estimated using the Kaplan–Meier method. Pearson correlation coefficients were calculated to estimate linear dependence between the expression values of candidate genes.

#### Quantitative PCR with reverse transcription

Complementary DNA was synthesized using the SuperScript IV Reverse Transcriptase Kit (catalog no. 18090-200; Invitrogen) according to the manufacturer’s instructions. quantitative PCR with reverse transcription (RT–qPCR) was performed for the genes of interest and two housekeeping genes using the Platinum SYBR Green qPCR Superix-UDG kit (catalog nos. 11733038/11733-046; Invitrogen). Primers are listed in Supplementary Table 2.

### Epigenetic characterization of tumors and cell lines

#### Chromatin immunoprecipitation followed by sequencing analysis of histone modifications in neuroblastoma primary tumors and cell lines

Formaldehyde cross-linking of cells, cell lysis, sonication, chromatin immunoprecipitation and library preparation were performed as described previously^53^, starting with approximately 4 × 10^6^ cells (1 × 10^6^ cells per individual immunoprecipitation). Direct cell lysis for each sample was achieved by 30 min incubation on ice in 950 µl radioimmunoprecipitation assay I buffer using approximately 30 mg of fresh-frozen tumor tissue per individual chromatin immunoprecipitation followed by sequencing (ChIP–seq) experiment. Library preparation was performed using the NEBNext Ultra DNA Library Prep Kit (New England Biolabs) according to the manufacturer’s protocol. Samples were mixed in equal molar ratios and sequenced on an Illumina sequencing platform.

#### ChIPmentation of MYCN transcription factor in neuroblastoma cell lines

Formaldehyde cross-linking, cell lysis, sonication and chromatin immunoprecipitation were performed as described previously^6^, adding the ChIPmentation module by Schmidl et al.^54^ with the following changes: a Bioruptor Plus with automated cooling (4 °C) was used for high-intensity sonication (20–30 min each with 30 s on and 30 s off intervals) and 10 µg MYCN antibody and 10^6^ cells for ChIP. The tagmentation reaction (Illumina Nextera DNA library Prep Kit) was performed at 37 °C for 1 min with the bead-bound chromatin sample or 5 ng purified input DNA for normalization. After de-cross-linking, purified samples were amplified using the dual index barcodes of the Nextera Index Kit and 13 PCR cycles. Enriched libraries were purified and pooled. ChIPmentation libraries were sequenced (50 single-end bases) on the Illumina sequencing platform (German Cancer Research Center Core Facility).

#### Data analysis of ChIP–seq and ChIPmentation

Single-end reads were aligned to the hg19 genome using Bowtie2 v.2.3.0, keeping uniquely aligned reads only. The BAM files of aligned reads were further processed using the deepTools suite v.3.0 (ref. ^55^). Input files were subtracted from the treatment files using the bamCompare tool v.3.0, applying the simple exponential smoothing method to normalize signal to noise. The resulting signals were normalized to an average 1× coverage to produce signal (bigWig) files. Peaks were called using the MACS2 v.2.1 tool using default parameters. Data are available at the Gene Expression Omnibus (GEO) under accession no. GSE189174.

#### DNA methylation analysis

DNA methylation and gene expression data from 105 primary neuroblastomas assessed by Infinium HumanMethylation450 BeadChips and 44K Agilent oligonucleotide microarrays^56^ (GEO accession no. GSE73518) were analyzed for candidate loci. The R2: Genomics Analysis and Visualization Platform (http://r2.amc.nl) was used to visualize the expression/methylation of selected gene–CpG pairs.

#### Tumor proteome analysis

Tumor proteome data were generated as part of a previous study^7^ and was reanalyzed for this study. Briefly, tumor samples were lysed in SDS, homogenized, split into replicates, reduced, alkylated and purified by Wessel-Flügge precipitation. Samples were then digested by LysC and trypsin and fractionated by strong cation exchange before being measured by reversed phase liquid chromatography–mass spectrometry (LC–MS) on Q Exactive Plus instruments (Thermo Fisher Scientific). Proteins quantified in less than 50% of MYCN high- or low-risk cases were excluded. Data were imputed by random draw from a normal distribution with default parameters: 0.3 width and 1.8 downshift. A two-sided Welch’s *t*-test was used to calculate the *P* values for the differential protein expression analysis; multiple testing correction by the Benjamini–Hochberg method was applied.

### Statistics and reproducibility

No statistical method was used to predetermine sample size. No data were explicitly excluded from the analyses unless they were of poor quality as determined by standard sequencing quality control metrics. The experiments were not randomized and the investigators were not blinded to allocation during the experiments and outcome assessment. Data are presented as the mean ± s.e.m. Statistical analyses were performed using Prism 7 (GraphPad Software). A two-tailed unpaired or paired Student’s *t*-test was used for comparisons between two groups. For the animal experiments, sample size was calculated with the help of a biostatistician using R v.3.4.0. Assumptions for the power analysis were as follows: Alpha error, 5%; Beta error, 20%. Mice were randomized into treatment groups before treatment. In case animals had to be euthanized before the predefined end point (due to weight loss or other termination criteria), they were excluded from any downstream analyses. All animal experiments (apart from animal treatment) were blinded during the experiments and follow-up assessment.

### Reporting Summary

Further information on research design is available in the Nature Research Reporting Summary linked to this article.

## Supplementary information

Reporting Summary

Supplementary Tables Supplementary Table 1: List of top hits from the MYCN synthetic lethal high-troughput screen. Supplementary Table 2: gRNAs and oligonucleotides used in the paper.

## Data Availability

Proteome data of neuroblastoma tumors were previously published by Hartlieb et al.^7^. All data were deposited with the European Genome-phenome Archive (as dataset no. EGAD00001006737) as part of study no. EGAS00001004349. Data are available upon request by contacting F.W. The RNA-seq data of 498 primary neuroblastoma patients were previously published^38^ and are available at the GEO under the accession no. GSE49711. The DNA methylation data of primary neuroblastoma tumors were previously published^18^ and are available at the GEO under the accession no. GSE73518. Time course RNA-seq profiling of IMR5/75 high MYCN and low MYCN cells was previously published^11^ and can be accessed at the GEO under the accession no. GSE97774. ChIP–seq data were deposited at the GEO under the accession no. GSE189174. The aligned BAM files of the RNA expression profiles of the high MYCN depletion/rescue experiments were submitted to the European Nucleotide Archive and can be found under accession no. PRJEB25184. The data of the MYCN synthetic lethal siRNA screen has been added to this article as supplementary data. The microarray data from tumor samples were deposited with the GEO under accession no. GSE192976. The remaining data are available within the article, supplementary information or from the corresponding authors upon reasonable request. Source data are provided with this paper.

## Source data

Source Data Fig. 1 Statistical source data.
Source Data Fig. 1 Unprocessed western blots.
Source Data Fig. 2 Statistical source data.
Source Data Fig. 2 Unprocessed western blots.
Source Data Fig. 3 Statistical source data.
Source Data Fig. 3 Unprocessed western blots.
Source Data Fig. 4 Statistical source data.
Source Data Fig. 5 Statistical source data.
Source Data Fig. 5 Unprocessed western blots.
Source Data Fig. 6 Statistical source data.
Source Data Fig. 7 Statistical source data.
Source Data Extended Data Fig. 1 Statistical source data.
Source Data Extended Data Fig. 1 Unprocessed western blots.
Source Data Extended Data Fig. 2 Statistical source data.
Source Data Extended Data Fig. 2 Unprocessed western blots.
Source Data Extended Data Fig. 3 Statistical source data.
Source Data Extended Data Fig. 3 Unprocessed western blots.
Source Data Extended Data Fig. 4 Statistical source data.
Source Data Extended Data Fig. 5 Statistical source data.
Source Data Extended Data Fig. 6 Statistical source data.

## Code availability

The custom scripts used to analyze the RNA-seq, microarray and ChIP–seq data are available upon request.
