# Supplementary material for: MYCN mediates cysteine addiction and sensitizes neuroblastoma to ferroptosis
Source: Nat Cancer. 2022 Apr 28;3(4):471–85. doi: 10.1038/s43018-022-00355-4 (PMC9050595; doi:10.1038/s43018-022-00355-4)
Supplement: Supplementary file 1 — Reporting Summary [file 43018_2022_355_MOESM1_ESM.pdf]

nature portfolio

Corresponding author(s): Hamed Alborzinia, Frank Westermann

Last updated by author(s): Dec 12, 2021

Reporting Summary

Nature Portfolio wishes to improve the reproducibility of the work that we publish. This form provides structure for consistency and transparency in reporting. For further information on Nature Portfolio policies, see our Editorial Policies and the Editorial Policy Checklist.

Statistics

For all statistical analyses, confirm that the following items are present in the figure legend, table legend, main text, or Methods section.

n/a	Confirmed	
☐	☒	The exact sample size (*n*) for each experimental group/condition, given as a discrete number and unit of measurement
☐	☒	A statement on whether measurements were taken from distinct samples or whether the same sample was measured repeatedly
☐	☒	The statistical test(s) used AND whether they are one- or two-sided<br>*Only common tests should be described solely by name; describe more complex techniques in the Methods section.*
☐	☒	A description of all covariates tested
☐	☒	A description of any assumptions or corrections, such as tests of normality and adjustment for multiple comparisons
☐	☒	A full description of the statistical parameters including central tendency (e.g. means) or other basic estimates (e.g. regression coefficient) AND variation (e.g. standard deviation) or associated estimates of uncertainty (e.g. confidence intervals)
☐	☒	For null hypothesis testing, the test statistic (e.g. *F*, *t*, *r*) with confidence intervals, effect sizes, degrees of freedom and *P* value noted<br>*Give P values as exact values whenever suitable.*
☒	☐	For Bayesian analysis, information on the choice of priors and Markov chain Monte Carlo settings
☐	☒	For hierarchical and complex designs, identification of the appropriate level for tests and full reporting of outcomes
☐	☒	Estimates of effect sizes (e.g. Cohen's *d*, Pearson's *r*), indicating how they were calculated

Our web collection on statistics for biologists contains articles on many of the points above.

Software and code

Policy information about availability of computer code

Data collection

NA

Data analysis

Software used in the study:
R (version 3.4.0)
Image J (version 1.51)
FlowJo version 10.6.0
Microarray analysis (as described in Klaus & Reisenauer, F1000Res 2016)
Bioconductor - limma (version 3.46)
BHC in-house software (developed and owned by BAYER Health care, intellectual property)

RNAseq:
STAR (version 2.5.3a)
R (version 4.3.0 & 3.4.3)
R - edge R (version 3.20.9)
R - gplots (version 3.1.1)

ChIPseq:
trimgalore (version 0.4.3)
bowtie2 (version 2.3)
deeptools (version 3.0)
macs2 (version 2.1)

For manuscripts utilizing custom algorithms or software that are central to the research but not yet described in published literature, software must be made available to editors and reviewers. We strongly encourage code deposition in a community repository (e.g. GitHub). See the Nature Portfolio guidelines for submitting code & software for further information.

Data

Policy information about availability of data

All manuscripts must include a data availability statement. This statement should provide the following information, where applicable:

- Accession codes, unique identifiers, or web links for publicly available datasets
- A description of any restrictions on data availability
- For clinical datasets or third party data, please ensure that the statement adheres to our policy

All information about data availability is provided in the data availability statement of the article:

Proteome data of neuroblastoma tumors was previously published by (Hartlieb, S. A. et al. Nat Commun 2021).and all data is deposited at the European Genome-Phenome Archive (EGA) as dataset EGAD00001006737 as part of the study EGAS00001004349. Data is available upon request by contacting Frank Westermann. RNA-seq data of 498 primary neuroblastoma patients was previously published (Lutz, W. et al. 1996)and is available at Gene Expression Omnibus (GEO) under the accession number GSE62564. DNA methylation data of primary neuroblastoma tumors was previously published (Cassago, A. et al. 2012) and is available at GEO under the accession number GSE73518. Time-course RNAseq profiling of IMR5/75 MYCN-high and MYCN-low cells was previously published (Muth, D. et al. 2010). and can be accessed at GEO under the accession number GSE97774. ChIP-seq data is deposited at GEO under the accession number GSE189174. The aligned .bam files of RNA expression profiles of the high-MYCN depletion/rescue experiments were submitted to the European Nucleotide Archive (ENA) and can be found under the accession number PRJEB25184. Data of the MYCN synthetic lethal siRNA screen is added to this article as Supplementary Data. Microarray data from tumor samples is deposited in GEO under the accession number GSE192976. Source data are provided with this paper. The remaining data are available within the Article, Supplementary Information or available from the authors upon reasonable request.

Field-specific reporting

Please select the one below that is the best fit for your research. If you are not sure, read the appropriate sections before making your selection.

☒ Life sciences ☐ Behavioural & social sciences ☐ Ecological, evolutionary & environmental sciences

For a reference copy of the document with all sections, see nature.com/documents/nr-reporting-summary-flat.pdf

Life sciences study design

All studies must disclose on these points even when the disclosure is negative.

Sample size	For in vivo studies, sample size was calculated with the help of a biostatistician using R version 3.4.0. Assumptions for power analysis were as follows: α error, 5%; β error, 20%. Values for standard deviations and differences between experimental groups were based on previous experiments (whenever a similar data type was available). for in vitro studies we used at least 3 biological replicates for each experiments.
Data exclusions	no data were excluded
Replication	in vitro experiments were repeated at least three times with similar results.
Randomization	For in vivo experiments mice were randomized into treatment groups prior to treatment. In case animals had to be sacrificed before the pre-defined endpoint (due to weight loss or other termination criteria), they were excluded from any downstream analyses.<br>For the in vitro experiments, all samples were analyzed equally with no subsampling; therefore, there was no requirement for randomization
Blinding	For in vivo experiments all animal experiments (except during animal treatment) were blinded during entire experiments or follow up assessment. For in vitro studies, data collection and analysis were not performed blinded to the conditions of the experiments.

Reporting for specific materials, systems and methods

We require information from authors about some types of materials, experimental systems and methods used in many studies. Here, indicate whether each material, system or method listed is relevant to your study. If you are not sure if a list item applies to your research, read the appropriate section before selecting a response.

Materials & experimental systems

n/a	Involved in the study
☐	☒ Antibodies
☐	☒ Eukaryotic cell lines
☒	☐ Palaeontology and archaeology
☐	☒ Animals and other organisms
☐	☒ Human research participants
☒	☐ Clinical data
☒	☐ Dual use research of concern

Methods

n/a	Involved in the study
☐	☒ ChIP-seq
☐	☒ Flow cytometry
☒	☐ MRI-based neuroimaging

Antibodies

Antibodies used

MYCN (clone B8.4.B, sc-53993, Santa Cruz, Lot. B2316, 1:1000)
c-MYC (clone Y69, ab32072, Abcam, Lot. 625369, 1:1000)
CTH (ab54573, Abcam, Lot. GR3260298-2, 1:1000)
SAHH (A-11) (AHCY antibody, clone A-11, sc-271389, Santa Cruz, Lot. C2111, 1:1000),
GPX4 (ab41787, Abcam, Lot. GR56784-1, 1:1000),
CARS (clone EPR7121, ab126714, Abcam, Lot. YI081819DS, 1:1000)
Glutaminase 1 (clone EP7212, ab156876, Abcam, Lot. GR249636-29,1:40,000)
Loading control: vinculin (clone 749, sc-73614, Santa Cruz, Lot. A2319, 1:1000) or β-actin-conjugated (ab20272, Abcam, Lot. GR3418697-1, 1:5000)
Secondary AB: HSR–peroxidase labeled anti-mouse (115-035-003, Dianova, 1:1000) or anti-rabbit (111-035-144, Dianova, 1:1000).

ChIP-seq: For all ChIP-seq experiments 3μg of antibody were used per ChIP.
H3K27me3; rabbit polyclonal; Active Motif 39155; Lot.31014017
H3K36me3; rabbit polyclonal; Abcam ab9050; Lot.GR273250-1
H3K9me3; rabbit polyclonal; Abcam; ab8898; Lot.GR148830-2
H3K27ac; rabbit polyclonal; Abcam; ab4729; Lot.GR183919-2

Validation

All antibodies used in this study were validated by manufactures for the species and specific application. Relevant validation results can be found in the website of each manufacture. A protein size marker was run on every western blot and the size of the assessed bands was compared to the manufactures information (See source data)

Eukaryotic cell lines

Policy information about cell lines

Cell line source(s)

Human neuroblastoma cell lines: IMR5/75, KELLY, SiMa, NBL-S, SK-N-FI, SH-SY5Y, NB69, SKNDZ, SH-EP, GI-ME-N.

SK-N-FI, SK-N-DZ, SH-SY5Y cells were purchased from ATCC.

KELLY, SiMa and GI-ME-N were purchased from DSMZ .

NB69 were kindly provided by Larissa Savelyeva

NBL-S and TET21N (SH-EP) were provided by G.M. Brodeur and W. Lutz, respectively.

Tunable cell lines, IMR5/75 MYCN shRNA, and SH-EP MYCN transgene (Tet21N) were generated and cultured as described previously (please see Methods section)

Authentication

Cell line identity/unique SNP profiles were confirmed by the Multiplexion Multiplex Cell Authentication service (Heidelberg, Germany). The purity of cell lines was validated using the Multiplex cell Contamination Test by Multiplexion (Heidelberg, Germany).

Mycoplasma contamination

All cell lines tested negative for mycoplasma contamination as noted in 'Materials and Methods'

Commonly misidentified lines
(See ICLAC register)

no commonly misidentified cell lines were used here

Animals and other organisms

Policy information about studies involving animals; ARRIVE guidelines recommended for reporting animal research

Laboratory animals

Mouse strains used in the study: NOD.Cg-PrkdcscidIl2rgtm1Wjl/SzJ (NSG, JAX stock #005557). Female mice, 3 – 4 months of age, were used for experiments. Mice were housed in individually ventilated cages under temperature and humidity control. Cages contained an enriched environment with bedding material.

March 2021

Wild animals	No wild animals were used in the study.
Field-collected samples	No wild animals were used in the study.
Ethics oversight	All studies involving mice and experimental protocols were conducted in compliance with German Cancer Center Institute guidelines and approved by the governmental review board of the state of Baden-Wuerttemberg, Regierungspraesidium Karlsruhe, under the authorization number G-176/19, followed the German legal regulations. Animals health were monitored daily and mice were euthanized as soon as they reached abortion criteria defined in the procedure

Note that full information on the approval of the study protocol must also be provided in the manuscript.

Human research participants

Policy information about studies involving human research participants

Population characteristics	Tumor samples are from patients enrolled in the German Neuroblastoma Trials of the GPOH (NB97, NB2004, NB2016). Detailed patient specific information (Age, Status, Gender etc) is provided in the previous publication by Gartlgruber et al, Nature Cancer 2020.
Recruitment	Almost all childhood neuroblastoma in Germany (>99%) are enrolled in a clinical trial, informed consent is given for the use of tumor material for research purposes.
Ethics oversight	All neuroblastoma patients were enrolled in the German Neuroblastoma Trial (NB97, NB2004, NB 2016) approved by the Ethics Committee of the University of Cologne and informed written consent was obtained from the patients' parents.

Note that full information on the approval of the study protocol must also be provided in the manuscript.

Clinical data

Policy information about clinical studies

All manuscripts should comply with the ICMJE guidelines for publication of clinical research and a completed CONSORT checklist must be included with all submissions.

Clinical trial registration	not applicable
Study protocol	not applicable
Data collection	not applicable
Outcomes	not applicable

Dual use research of concern

Policy information about dual use research of concern

Hazards

Could the accidental, deliberate or reckless misuse of agents or technologies generated in the work, or the application of information presented in the manuscript, pose a threat to:

No	Yes	
☒	☐	Public health
☒	☐	National security
☒	☐	Crops and/or livestock
☒	☐	Ecosystems
☒	☐	Any other significant area

Experiments of concern

Does the work involve any of these experiments of concern:

No	Yes	
☒	☐	Demonstrate how to render a vaccine ineffective
☒	☐	Confer resistance to therapeutically useful antibiotics or antiviral agents
☒	☐	Enhance the virulence of a pathogen or render a nonpathogen virulent
☒	☐	Increase transmissibility of a pathogen
☒	☐	Alter the host range of a pathogen
☒	☐	Enable evasion of diagnostic/detection modalities
☒	☐	Enable the weaponization of a biological agent or toxin
☒	☐	Any other potentially harmful combination of experiments and agents

ChIP-seq

Data deposition

☒ Confirm that both raw and final processed data have been deposited in a public database such as GEO.

☒ Confirm that you have deposited or provided access to graph files (e.g. BED files) for the called peaks.

Data access links
May remain private before publication.

ChIP-seq data has been uploaded to GEO under the accessions GSE189174.

Files in database submission

GSM5695680 GI-ME-N H3K27ac
GSM5695681 GI-ME-N H3K27me3
GSM5695682 GI-ME-N H3K36me3
GSM5695683 GI-ME-N H3K4me1
GSM5695684 GI-ME-N H3K4me3
GSM5695685 GI-ME-N H3K9me3
GSM5695686 GI-ME-N input DNA
GSM5695687 IMR575 H3K27ac
GSM5695688 IMR575 H3K27me3
GSM5695689 IMR575 H3K36me3
GSM5695690 IMR575 H3K4me1
GSM5695691 IMR575 H3K4me3
GSM5695692 IMR575 H3K9me3
GSM5695693 IMR575 input DNA
GSM5695694 Kelly H3K27ac
GSM5695695 Kelly H3K27me3
GSM5695696 Kelly H3K36me3
GSM5695697 Kelly H3K4me1
GSM5695698 Kelly H3K4me3
GSM5695699 Kelly H3K9me3
GSM5695700 Kelly input DNA
GSM5695701 NB69 H3K27ac
GSM5695702 NB69 H3K27me3
GSM5695703 NB69 H3K36me3
GSM5695704 NB69 H3K4me1
GSM5695705 NB69 H3K4me3
GSM5695706 NB69 H3K9me3
GSM5695707 NB69 input DNA
GSM5695708 NBL-S H3K27ac
GSM5695709 NBL-S H3K27me3
GSM5695710 NBL-S H3K36me3
GSM5695711 NBL-S H3K4me1
GSM5695712 NBL-S H3K4me3
GSM5695713 NBL-S H3K9me3
GSM5695714 NBL-S input DNA
GSM5695715 SH-EP H3K27ac
GSM5695716 SH-EP H3K27me3
GSM5695717 SH-EP H3K36me3
GSM5695718 SH-EP H3K4me1
GSM5695719 SH-EP H3K4me3
GSM5695720 SH-EP H3K9me3
GSM5695721 SH-EP input DNA
GSM5695722 SK-N-FI H3K27ac
GSM5695723 SK-N-FI H3K27me3
GSM5695724 SK-N-FI H3K36me3

GSM5695725	SK-N-FI H3K4me1
GSM5695726	SK-N-FI H3K4me3
GSM5695727	SK-N-FI H3K9me3
GSM5695728	SK-N-FI input DNA
GSM5695729	SKNDZ H3K27ac
GSM5695730	SKNDZ H3K27me3
GSM5695731	SKNDZ H3K36me3
GSM5695732	SKNDZ H3K4me1
GSM5695733	SKNDZ H3K4me3
GSM5695734	SKNDZ H3K9me3
GSM5695735	SKNDZ input DNA

Genome browser session (e.g. UCSC)

No longer applicable.

Methodology

Replicates

For each tumor and cell line one biological replicate was done due to the limited amount of material.

Sequencing depth

ChIP-sequencing was done using Illumina HiSeq2000 50 bp single end sequencing.

Antibodies

H3K27me3; rabbit polyclonal; Active Motif 39155; Lot.31014017
H3K36me3; rabbit polyclonal; Abcam ab9050; Lot.GR273250-1
H3K9me3; rabbit polyclonal; Abcam; ab8898; Lot.GR148830-2
H3K27ac; rabbit polyclonal; Abcam; ab4729; Lot.GR183919-2

Peak calling parameters

Peak calling was performed with the MACS2 algorithm, both with the narrowPeak and broadPeak parameters; the FDR cutoff for peak calling was set to 1%.

Data quality

Only uniquely aligned reads are considered, and duplicates are removed.
Data quality was ensured both by visual inspection of reference region in the genome browser. Moreover, we assessed the enrichment signal using the fingerprint method developed as part of the DeepTools package (Diaz et al.), and using criteria recommended by the ENCODE consortium such as the PCR bottleneck coefficient and the FRiP (fraction of reads in peaks).

Software

The ChIP-seq processing was performed using a custom pipeline written in Snakemake; the steps involve (1) read trimming using TrimGalore, (2) alignment using Bowtie2 on the hg19 genome with standard parameters, (3) merginf of replicates if available, (4) peak calling using MACS2, (5) QC using the fingerprint and FRiP method, (6) SES normalization and bigwig generation by subtracting normalized input from IP.

Flow Cytometry

Plots

Confirm that:

☒ The axis labels state the marker and fluorochrome used (e.g. CD4-FITC).

☒ The axis scales are clearly visible. Include numbers along axes only for bottom left plot of group (a 'group' is an analysis of identical markers).

☒ All plots are contour plots with outliers or pseudocolor plots.

☒ A numerical value for number of cells or percentage (with statistics) is provided.

Methodology

Sample preparation

MYCN-low populations were established by incubating cells with 1 µg/ml doxycycline at least 48 h prior to further treatment. Cells were harvested and lipid peroxidation was analyzed, using C11-BODIPY BD of 4 µM final concentration in Hanks' Balanced Salt Solution (HBSS). Cells were incubated at 37°C for 15 min and signal intensity was measured. Total intracellular ROS levels were determined using CellROX® (according to Thermo Fisher Scientific instruction).

Instrument

MACSquant VYB, model number: 130-096-116, SN:3050
BD FACS Aria™ cell sorter IIu, model number: 355119, SN: P0087

Software

FlowJo software version 10.6.0 (commercial standard software for analysis of flow cytometric data).

Cell population abundance

In this manuscript, only one pure population at the time was analyzed (neuroblastoma cell lines).

Gating strategy

For all samples, an initial manual gate in SSC-A by FSC-A was set to identify live cells and exclude debris. From the live cells, a rectangular gate was set on FSC-H by FSC-A to exclude doublets, meaning cells off the diagonal. If DNA staining was carried out, an additional gate was set on DNA (Violet channel)-W by DNA-A to exclude additional cell debris or DNA doublets (Extended Data Figure 7))

☒ Tick this box to confirm that a figure exemplifying the gating strategy is provided in the Supplementary Information.
