# Supplementary material for: MYCN mediates cysteine addiction and sensitizes neuroblastoma to ferroptosis
Source: Nat Cancer. 2022 Apr 28;3(4):471–85. doi: 10.1038/s43018-022-00355-4 (PMC9050595; doi:10.1038/s43018-022-00355-4)

Figure 1a: Unprocessed Western Blot upon MYCN doxycyline(DOX_72 h)-induced shRNA-mediated knockdown (stable MYCNshRNA inducible knockdown NB IMR5/75 cell model)

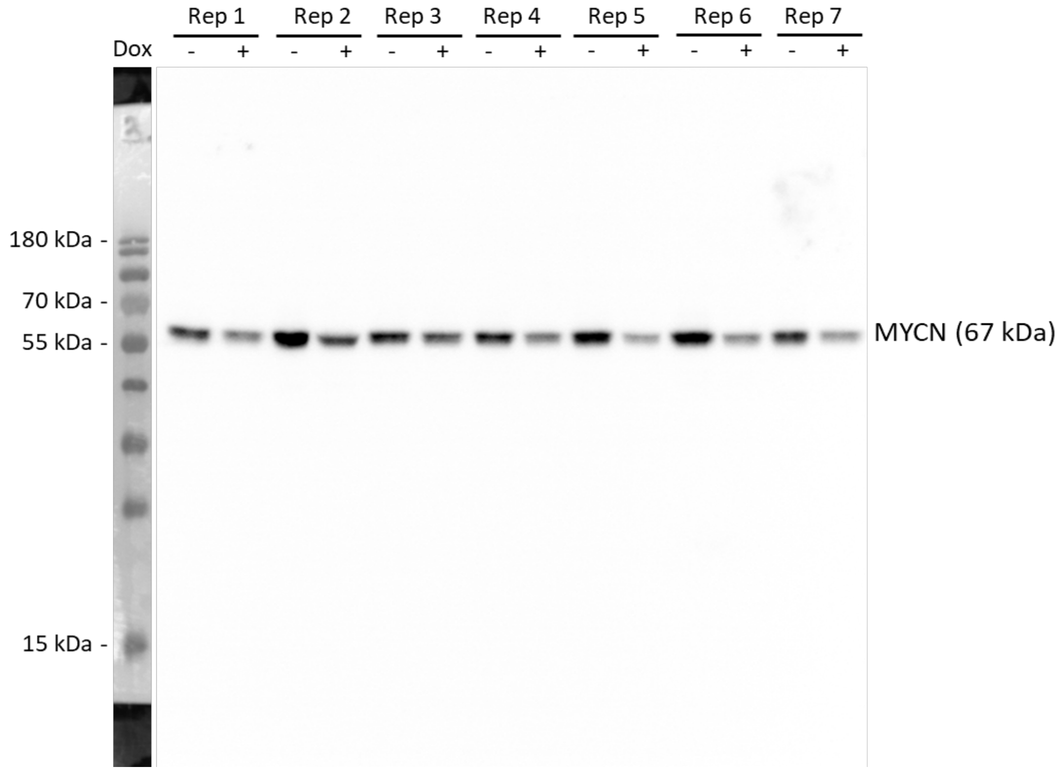

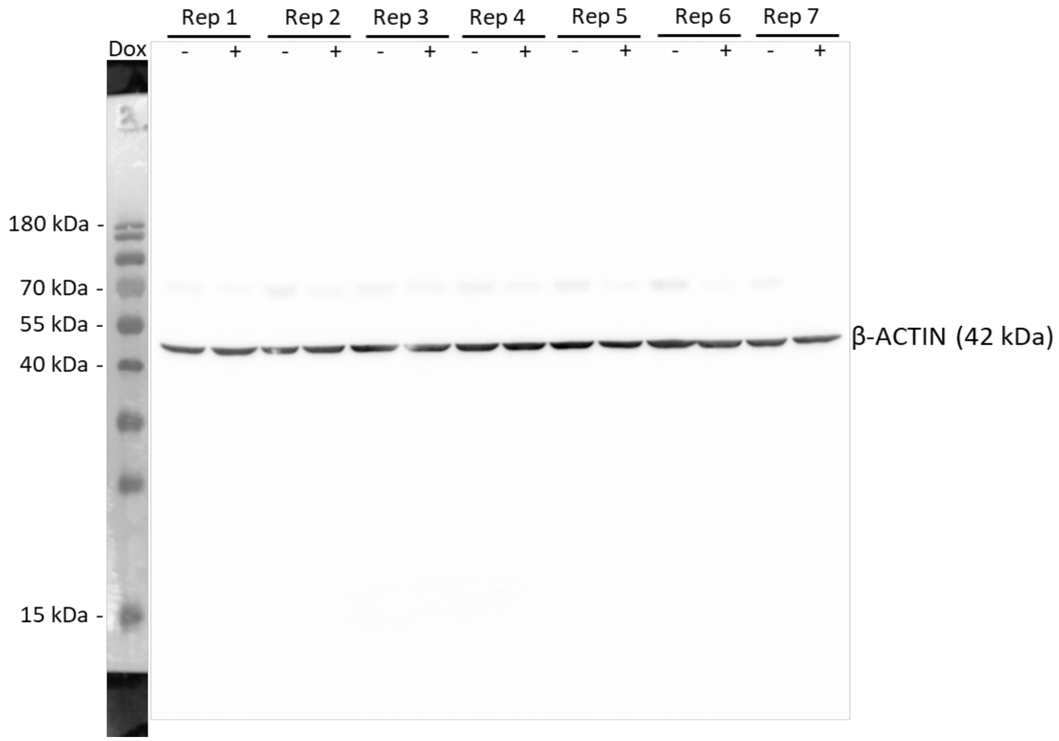

Figure 1e: Unprocessed Western Blot upon MYCN doxycyline(DOX_72 h)-induced shRNA-mediated knockdown (stable MYCNshRNA inducible knockdown NB TET21N cell model)

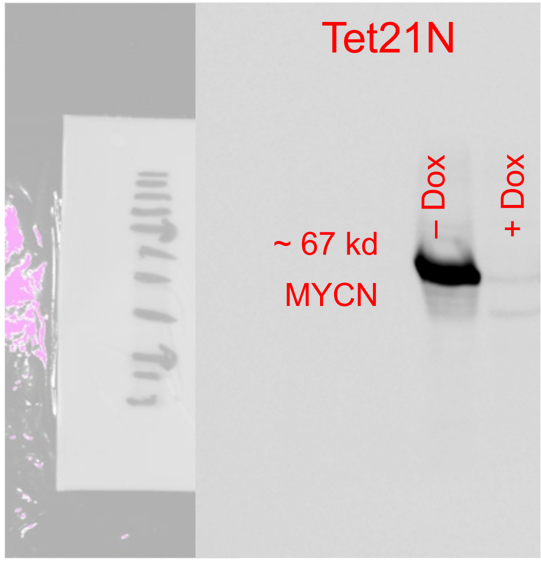

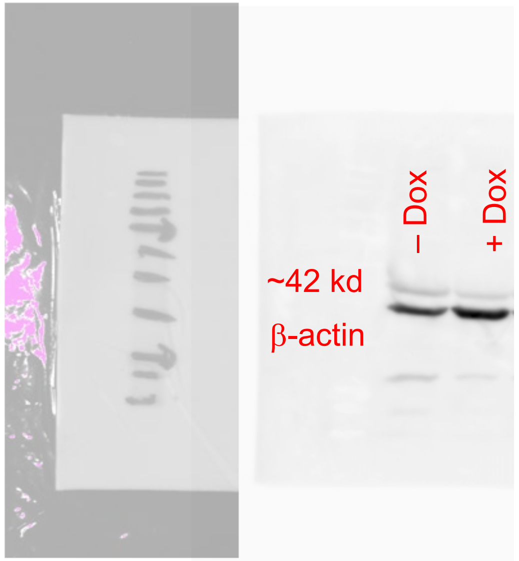

Supplement: Source Data Fig. 1 — Unprocessed western blots. [file 43018_2022_355_MOESM4_ESM.pdf]
