# Supplementary material for: MYCN mediates cysteine addiction and sensitizes neuroblastoma to ferroptosis
Source: Nat Cancer. 2022 Apr 28;3(4):471–85. doi: 10.1038/s43018-022-00355-4 (PMC9050595; doi:10.1038/s43018-022-00355-4)

Figure 3i: Unprocessed Western Blot upon GPX4 doxycyline(DOX)-induced CRISPR-mediated knockout in SKNDZ cell line in vitro

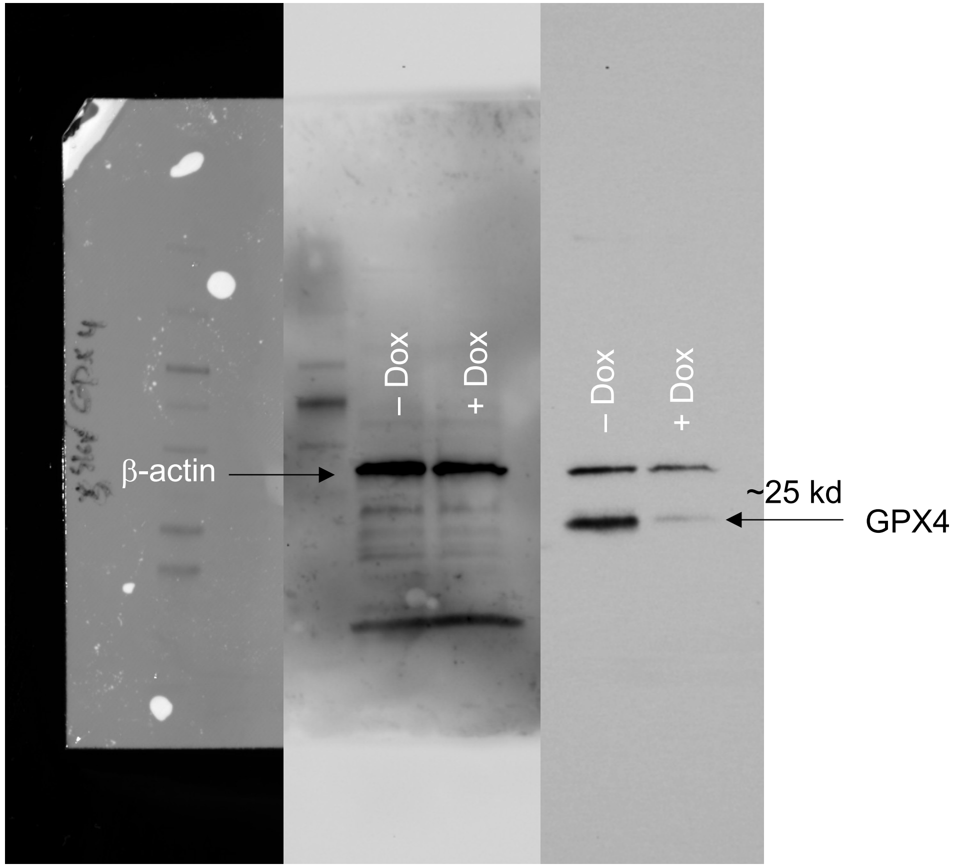

Figure 3h: Unprocessed Western Blot upon GPX4 doxycyline(DOX)-induced CRISPR-mediated knockout in SKNDZ in tumor samples

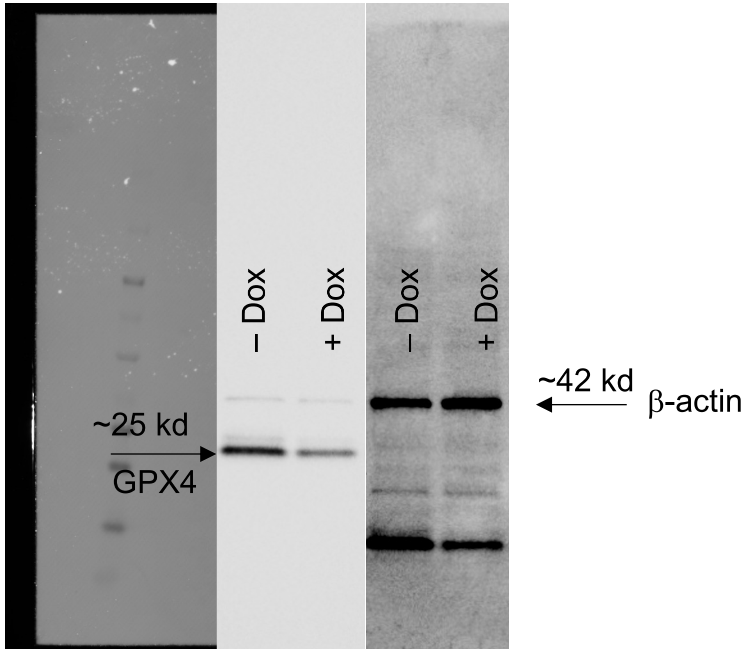

Supplement: Source Data Fig. 3 — Unprocessed western blots. [file 43018_2022_355_MOESM8_ESM.pdf]
