# Supplementary material for: MYCN mediates cysteine addiction and sensitizes neuroblastoma to ferroptosis
Source: Nat Cancer. 2022 Apr 28;3(4):471–85. doi: 10.1038/s43018-022-00355-4 (PMC9050595; doi:10.1038/s43018-022-00355-4)

Figure 5f: Unprocessed Western Blot and Colony formation assay upon AHCY doxycyline-induced shRNA-mediated knockdown (two different stable AHCYshRNA inducible knockdown NB cell models)

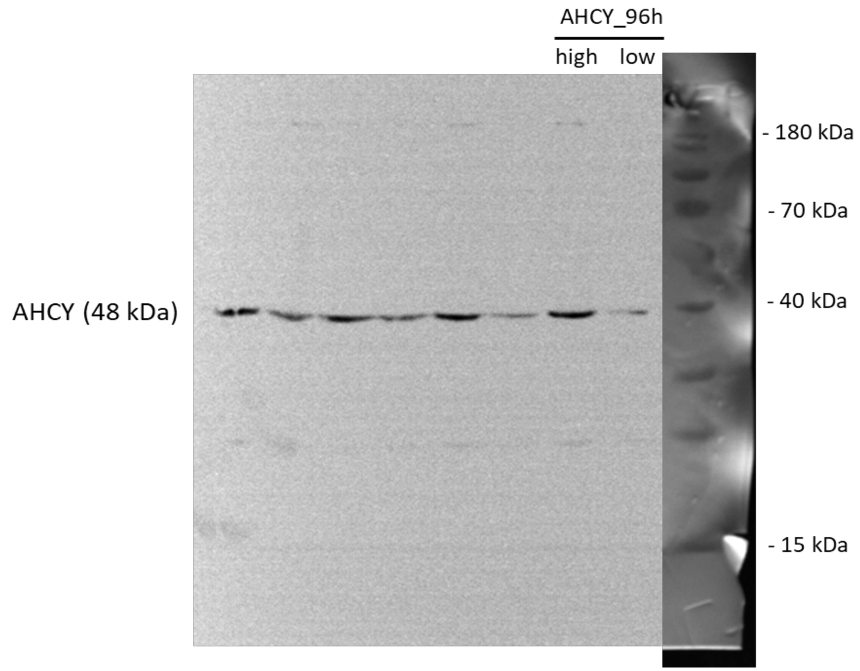

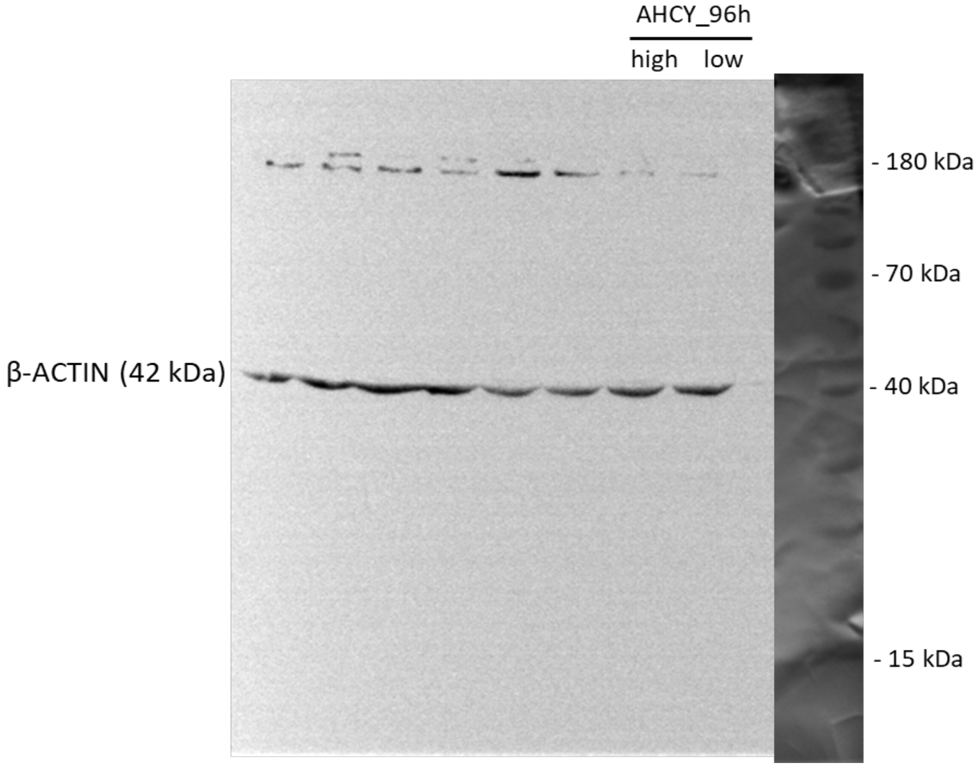

Figure 5h: Unprocessed Western Blot upon CARS siRNA-mediated knockdown (72 h CARS knockdown)

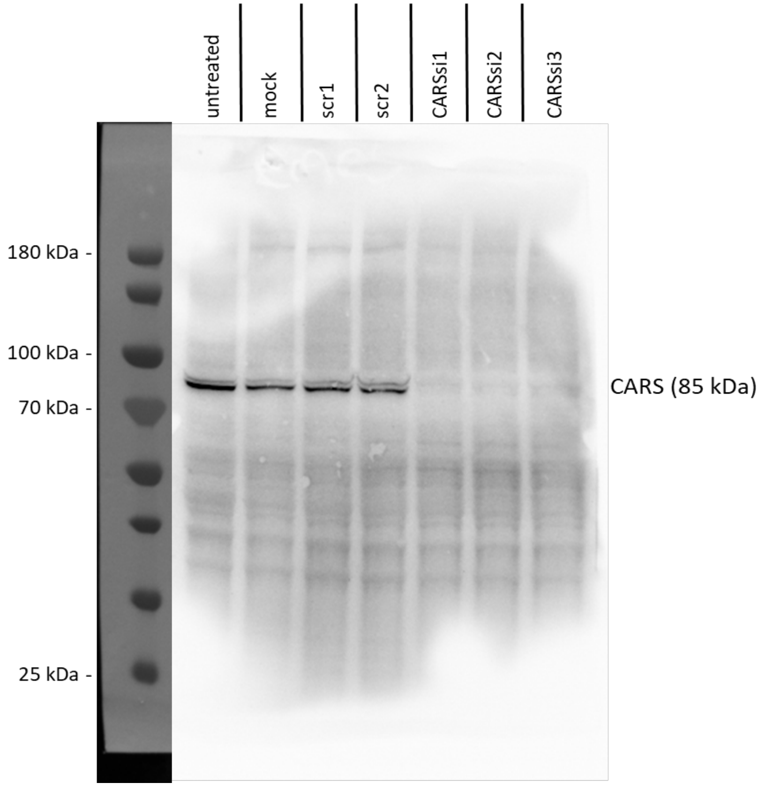

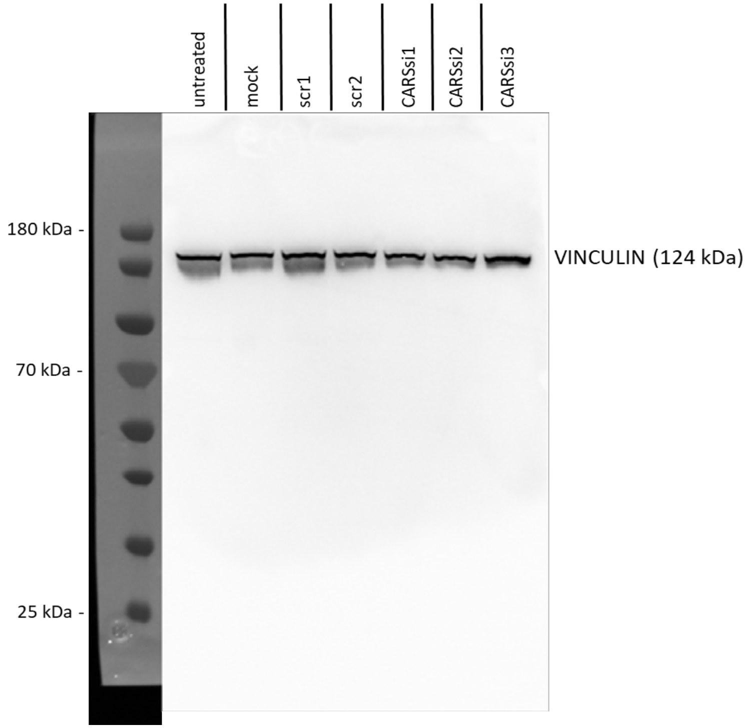

Supplement: Source Data Fig. 5 — Unprocessed western blots. [file 43018_2022_355_MOESM11_ESM.pdf]
