# Supplementary material for: MYCN mediates cysteine addiction and sensitizes neuroblastoma to ferroptosis
Source: Nat Cancer. 2022 Apr 28;3(4):471–85. doi: 10.1038/s43018-022-00355-4 (PMC9050595; doi:10.1038/s43018-022-00355-4)

Extended Data Figure 2b: Unprocessed Western Blot upon GPX4 siRNA-mediated knockdown (96 h GPX4 knockdown)

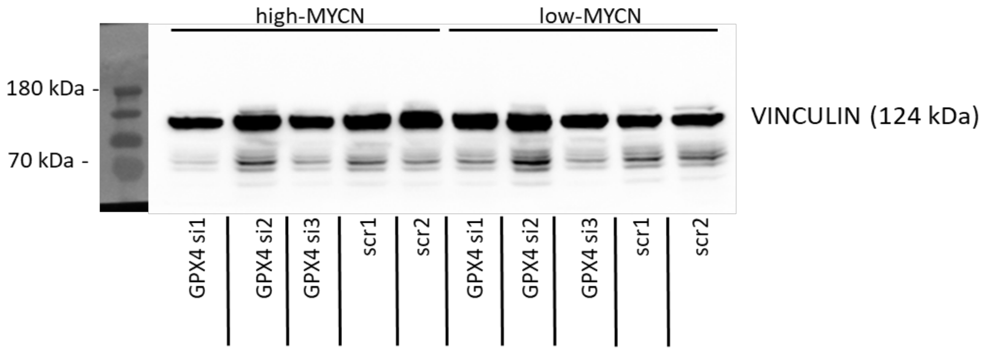

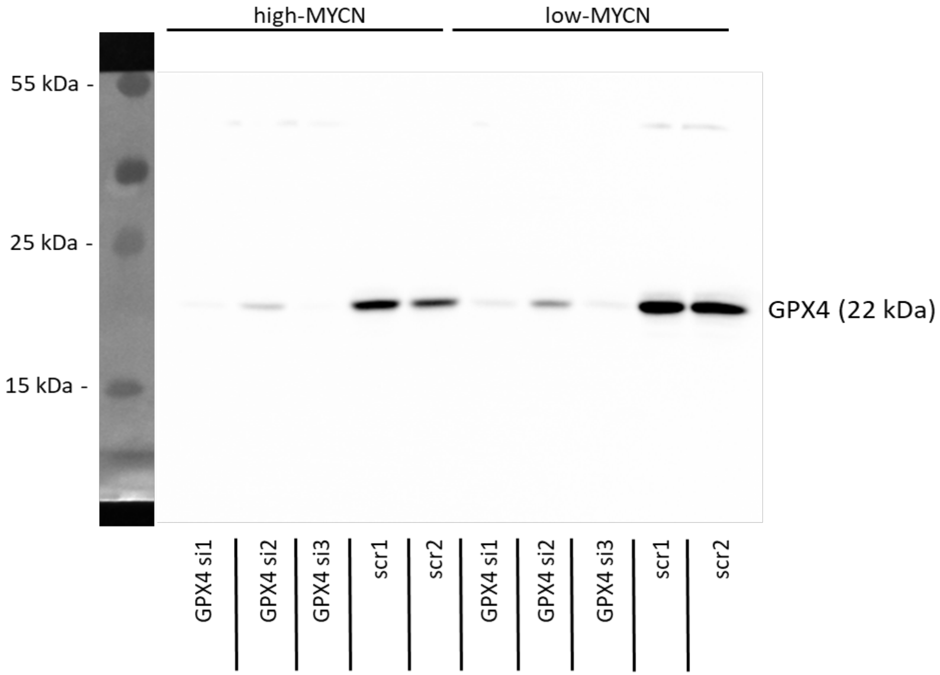

Supplement: Source Data Extended Data Fig. 2 — Unprocessed western blots. [file 43018_2022_355_MOESM17_ESM.pdf]
